# Origins of 1/f-like tissue oxygenation fluctuations in the murine cortex

**DOI:** 10.1371/journal.pbio.3001298

**Published:** 2021-07-15

**Authors:** Qingguang Zhang, Kyle W. Gheres, Patrick J. Drew

**Affiliations:** 1 Center for Neural Engineering, Department of Engineering Science and Mechanics, The Pennsylvania State University, University Park, Pennsylvania, United States of America; 2 Graduate Program in Molecular Cellular and Integrative Biosciences, The Pennsylvania State University, University Park, Pennsylvania, United States of America; 3 Department of Neurosurgery, The Pennsylvania State University, University Park, Pennsylvania, United States of America; 4 Department of Biomedical Engineering, The Pennsylvania State University, University Park, Pennsylvania, United States of America; University of Glasgow, UNITED KINGDOM

## Abstract

The concentration of oxygen in the brain spontaneously fluctuates, and the distribution of power in these fluctuations has a 1/f-like spectra, where the power present at low frequencies of the power spectrum is orders of magnitude higher than at higher frequencies. Though these oscillations have been interpreted as being driven by neural activity, the origin of these 1/f-like oscillations is not well understood. Here, to gain insight of the origin of the 1/f-like oxygen fluctuations, we investigated the dynamics of tissue oxygenation and neural activity in awake behaving mice. We found that oxygen signal recorded from the cortex of mice had 1/f-like spectra. However, band-limited power in the local field potential did not show corresponding 1/f-like fluctuations. When local neural activity was suppressed, the 1/f-like fluctuations in oxygen concentration persisted. Two-photon measurements of erythrocyte spacing fluctuations and mathematical modeling show that stochastic fluctuations in erythrocyte flow could underlie 1/f-like dynamics in oxygenation. These results suggest that the discrete nature of erythrocytes and their irregular flow, rather than fluctuations in neural activity, could drive 1/f-like fluctuations in tissue oxygenation.

## Abstract 2

Brain hemodynamic signals show "1/f-like" dynamics, but their relationship to neural activity is unclear. Using experimental and computational approaches, this study reveals that 1/f-like dynamics in brain oxygenation do not reflect neuronal activity, but instead may be explained by heterogeneity in red blood cells.

## Introduction

Fluctuations in oxygen tension are ubiquitous throughout the body and are found in muscle tissue and tumors [1], in the retina [2,3], in the carotid artery [4], and in the cortex [5–12]. Despite their ubiquity, relatively little is understood about the origin of these oxygen fluctuations. While some of these fluctuations are driven by fluctuations in respiration, such as the breathing rate and intensity [4,13–20], fluctuations in oxygen concentration are present covering a wide range of frequency, not just at the respiration frequency, with most of the power concentrated at lower (<0.1 Hz) frequencies [1,2,5,7–9]. The power spectrum of oxygen concentrations in many tissues shows a “1/f-like” behavior, that is, the power at any given frequency *f* is proportional to 1/*f*^*β*^, where the exponent *β* is usually between 1 and 2 [8]. The hallmark of 1/f-like signals is that the power at lower frequencies is much larger than at higher frequencies, producing signals with rapid, small oscillations riding on top of much larger, but slower fluctuations. We refer to these oscillations as being 1/f-like because they are only characterized within a limited frequency region (here, ≥0.01 Hz and ≤1 Hz). While many biological processes have been shown to exhibit 1/f-like dynamics, a process can only be said to be 1/f if there are data over at least 2 orders of magnitude in both the abscissa and ordinate [21], a criterion that only a few studies meet [8]. In contrast, white noise has a constant power across frequencies, which when fitted with a power law gives a *β* close to 0 (**S1 Fig**, panel A). In both cases, there can be “extra” spectral power concentrated in a single band, leading to a “bump” in the spectrum (**S1 Fig**, panels B and D). Measurements of brain tissue oxygenation in primates show a clear, statistically robust 1/f-like power spectra, with an additional peak near 0.1 Hz [8].

Brain tissue oxygenation is determined by the balance between the oxygen supplied by the blood and the oxygen consumed by mitochondria in neurons, astrocytes, and mural cells of the brain parenchyma. Both of these processes could contribute to fluctuations in oxygenation. Increases in brain neural activity are usually accompanied by vasodilation and increased blood flow/volume that leads to increases in oxygenation [22]. The resulting change in oxygenation will involve an interplay of factors, with the increase in blood flow usually, but not always, driving an oxygen increase [20]. The linkage of oxygenation to neural activity is widely used to infer neural activity noninvasively using blood oxygenation level-dependent (BOLD) functional magnetic resonance imaging (fMRI) [23]; however, there are many examples of neural and vascular signals departing from this relationship [24–29]. Converging evidence from a large body of studies in both rodents and primates have shown that power in the gamma band (nominally 40 to 100 Hz) of the local field potential (LFP) is most closely related to the vasodilation that leads to increased blood volume and flow [30–36]. Spiking activity has similar correlations to blood volume as gamma-band LFP power [30,37,38], while the correlations for other bands of the LFP are much lower [30,31,34]. The signal in the LFP is the sum of population activity within the spatial area spanned by the electrodes [39]. Its precise relations to underlying neuronal activity is complex [40], but the LFP is primarily driven by synaptic currents generated by the interaction between pyramidal neurons and parvalbumin-positive interneurons [41–43]. The synaptic currents that drive the LFP are largely generated by local spiking, not from input from other areas, as localized increase in pyramidal neuron activity (generated with optogenetic or chemogenetic approaches) causes large increases in gamma-band power [41,42,44], and suppression of local neural activity drives large decreases in gamma-band power [44]. Given the interrelatedness of gamma-band oscillations and local neuronal spiking, it is not surprising that in the awake animal, increases in local spiking and gamma-band power tend to be strongly correlated [45–48].

There have been speculations that the ultraslow (<1 Hz) electrical signals are the neural correlate of brain hemodynamics [49–51], but frequencies below 1 Hz in the LFPs are of a nonneuronal origin (see [36] for review; [52–57]). Because the electrical potential of the blood is negative relative to that of the cerebral spinal fluid [52,53], changes in the blood volume in the brain will generate ultraslow potentials. The dilation of arterioles (occurs over seconds) and veins (occurs over tens of seconds) in awake animals’ brain [58–60] will generate changes (<1 Hz) in the LFP [54–57]. The nonneuronal origin of <1 Hz electrical signals has been shown with manipulations that dilate or constrict blood vessels independent of changes in neural activity, such as CO_2_ inhalation [55–57,61], head tilt, and Valsalva maneuver [54]. Additionally, most amplifiers have circuitry setup to reject these very low frequencies [62], so unless the recording setup is specifically designed to measure at DC frequencies, signals <1 Hz are not of a physiological origin.

Though there are many studies investigating the relationship between neural activity and vasodilation, there is a paucity of studies simultaneously measuring neural activity and oxygen changes [63–66], with only a handful looking in awake animals [8,20,67]. Whether 1/f-like dynamics in brain oxygenation are driven by neural activity bears on the interpretation of hemodynamic imaging. Several fMRI studies have suggested that 1/f-like dynamics exist in human BOLD signals [68–71], and the 1/f-like fluctuations in brain hemodynamics have been interpreted as being driven by 1/f-like fluctuations in neural activity [72,73]. However, recordings of the LFP in both humans [74] and nonhuman primates [75] do not seem to show 1/f dynamics in band-limited power (BLP).

As 1/f-like oxygen fluctuations are found in other organs besides the brain [1,2], their origin may not be neural and could come from vascular process. Blood flow and arterial diameter show fluctuations in a similar frequency range as oxygen fluctuations [3]. Additionally, as oxygen is carried by red blood cells (RBCs), fluctuations in the flux of RBCs can drive erythrocyte-associated transients (EATs) in oxygen in the tissue [76–92], and fluctuations in flux of these changes in local oxygenation in the cortex [93–97]. Stalls, brief stoppages in blood flow through capillaries, happen sporadically and continuously in the cortex due to transient blockage of blood flow by leukocytes [98–103], which are known to greatly increase vascular resistance [104]. These blockages likely drive changes in tissue oxygenation [105], and increased frequency of these stalls has been linked to neurodegenerative disorders [98,99,105].

To understand the relationship between neural activity and 1/f-like oxygen tension oscillations in the brain, we used oxygen polarography to directly measure brain tissue oxygenation in different cortical regions and layers in awake mice. We find that in unanesthetized, head-fixed mice, (1) cortical oxygenation showed 1/f-like power spectra that are similar across cortical regions and layers; (2) the BLP of LFP activity did not show 1/f-like power spectra; (3) there was significant coherence and correlation between neural activity and tissue oxygenation, but both were small; (4) silencing neural activity did not stop 1/f-like fluctuations in brain oxygenation; and (5) simulations of erythrocyte flow, taking into account the statistics of erythrocyte spacing, showed that the irregular nature of erythrocyte spacing can generate 1/f-like dynamics in tissue oxygenation. Our results suggest that the driver of 1/f-like oxygenation fluctuations is nonneuronal in origin and could be due to fluctuations in RBC flux through the capillary network.

## Results

We measured tissue oxygenation signals and neural activity from the somatosensory and frontal cortices of awake behaving mice head fixed on a spherical treadmill [20,24,30,106]. We recorded laminar neural activity with linear multisite probes in 7 mice, laminar oxygenation using polarographic electrodes in 37 mice, and simultaneous neural activity, respiration, and oxygen measurements in 9 mice. Additionally, 9 mice were used to measure RBCs spacing in capillaries using two-photon laser scanning microscopy (2PLSM). We reported results for “rest,” which only include data from periods of time when the animal was not locomoting, or for all data, which include periods of locomotion and rest. We did this because unanesthetized mice engage in spontaneous movement frequently, and these spontaneous movements are large drivers of neural activity and hemodynamic signals [30,36,107–109]. Specifically, cutaneous sensation during locomotion drives large increases in neural activity in the forelimb/hindlimb (FL/HL) region [20,24,110,111]. The increase in neural activity drives localized increases in blood flow, which is not due to systemic factors [20,112]. Neural and oxygen measurements were made in the frontal cortex (FC) and in the FL/HL region of the somatosensory cortex (identified by cytochrome oxidase staining [113]). All power spectra and frequency-domain analyses were done using multitaper techniques [114], which minimize spectral leakage, using the Chronux toolbox (http://chronux.org/). In addition, we applied a time-domain analysis method, detrended fluctuation analysis (DFA) [115], which complements the frequency-domain approach, to rigorously test the 1/f-like dynamics in various signals. Portions of this dataset have been published previously [20]. In this previous report, we found that locomotion significantly and globally increases cerebral oxygenation, in brain regions involved in locomotion, as well as in the FC and the olfactory bulb. The oxygenation increase persists when neural activity and functional hyperemia are blocked, occurred both in the tissue and in arteries feeding the brain, and is tightly correlated with respiration rate and the phase of respiration cycle.

### Brain oxygenation shows 1/f-like power spectrum with a band-limited component

**Fig 1:**
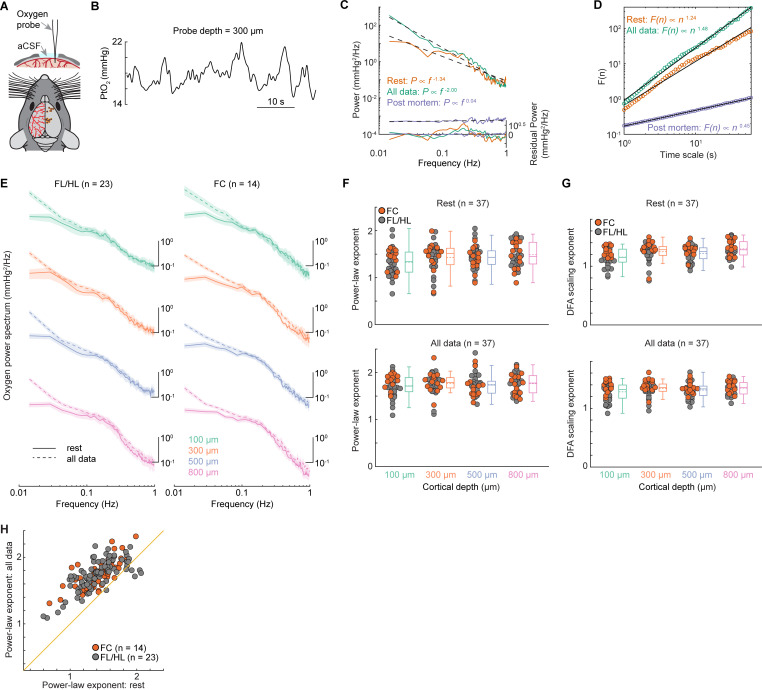
1/f-like dynamics of brain tissue oxygenation. (**A**) Experimental setup (top) and a summary of measurement sites (bottom). (**B**) Example trace showing the spontaneous fluctuations of tissue oxygenation (PtO_2_) 300 μm below the pia. (**C**) Representative power spectrum of the PtO_2_ (solid line) as well as its power law fit (dashed line) using data from resting period (orange) and periods including both rest and locomotion (green). The residual power (i.e., the difference between the power spectrum of the observed oxygen signal and the power law fit) is shown in the bottom. The purple trace indicates the power spectrum of PtO_2_ and its linear regression fit from a mouse postmortem. The power law exponent (−0.04) of the linear regression fit is reduced to near white noise. (**D**) Example showing the DFA scaling from the same datasets in (**C**). (**E**) Group average of PtO_2_ power spectrum during periods of rest (solid line) and periods including both rest and locomotion (dashed line) across different cortical depths in both FL/HL (*n =* 23 mice) and FC (*n =* 14 mice). Data are shown as mean ± SEM. For better visualization and comparison between these signals, the power spectrum curves have been vertically shifted between different cortical depths. (**F**) Group average (*n =* 37 mice) of power-law exponent across different cortical layers during periods of rest (top) and periods including both rest and locomotion (bottom). Gray circles denote the measurements in FL/HL (*n* = 23 mice), while the orange circles denote the measurements in FC (*n* = 14 mice). (**G**) As (**F**) but for DFA scaling exponents. (**H**) Scatter plot showing the increase of power-law exponent during periods including locomotion. The orange line indicates the unity line. Data in (**F**) and (**G**) are shown as median ± interquartile range using boxplot, with the sample mean shown as dashed lines. The data used to generate this figure are available at https://doi.org/10.5061/dryad.pg4f4qrmt. DFA, detrended fluctuation analysis; FC, frontal cortex; FL/HL, forelimb/hindlimb.

We first asked if tissue oxygen concentrations (PtO_2_) in the cortex of awake mice showed 1/f-like power spectra, as has been observed in the cortex of nonhuman primates [8]. We collected tissue oxygenation in multiple sites in 37 awake behaving mice (**Fig 1A**). The average duration of recording at each site was 37.0 ± 11.6 minutes. Examination of the resting PtO_2_ trace reveals that oxygen levels show slow fluctuations on the time scale of seconds or longer (**Fig 1B**). The power spectra of tissue oxygen signals, when plotted on a log–log axis, was linear, with a band-limited component (in the range of 0.1 to 0.3 Hz) (**Fig 1C**), as seen in nonhuman primates [8]. The band-limited oscillations cover the frequency band in which spontaneous arterial oscillations are seen in vivo when neural activity is blocked [30] and ex vivo in cannulated arteries [116–119]. As a control for any nonphysiological sources of this signal [120,121], we measured PtO_2_ in a mouse postmortem. The power spectrum of PtO_2_ in the mouse postmortem was essentially flat (with an exponent of −0.04; **Fig 1C**), characteristic of white noise (**S1 Fig**, panel A), and was several orders of magnitude smaller at all frequencies, ruling out a nonphysiological origin of these fluctuations.

We then quantified the nature of the power spectrum of oxygen fluctuations by fitting it with a power law distribution in the 0.01 to 1 Hz frequency range, since fitting of alternative models (an exponential model and a log-normal model) to the oxygen power spectrum does not provide significantly better fits for all the data (though in some cases, they were better fits for resting data; see **S1 Table** and **S1 Text**). To estimate the power-law exponent, we fitted the oxygen power spectrum using an ordinary least squares regression (see Methods), to allow comparisons to previous studies [8,68], though there are caveats to this approach [122]. The coefficient of determination (R^2^) of power law fits were high, both for resting data only (R^2^ = 0.62 ± 0.22) and using all the data (R^2^ = 0.96 ± 0.06). Averaged across all animals (*n =* 37), the power law exponent during rest was 1.42 ± 0.19, comparable to what have been observed in unanesthetized nonhuman primates (1.74) using oxygen-sensitive microelectrodes [8], but somewhat larger than those observed in human BOLD studies [68]. We then asked if the exponent of the fit to the power spectrum differed across cortical layers, since there are laminar differences in vascular, mitochondrial, and cellular density [123–125], which could affect the oxygen dynamics [95,96]. No significant differences of power-law exponents were observed among different cortical depths at rest (1.33 ± 0.29 at 100 μm, 1.43 ± 0.32 at 300 μm, 1.43 ± 0.25 at 500 μm, 1.49 ± 0.27 at 800 μm, *n =* 37 mice, **Fig 1F**, one-way ANOVA, F(3,143) = 1.9606, *p* = 0.1227), though resting PtO_2_ was lower at 100 μm compared to 300 μm, 500 μm, and 800 μm below the pia (12.68 ± 6.57 mm Hg at 100 μm, 20.50 ± 9.55 mm Hg at 300 μm, 21.58 ± 9.76 mm Hg at 500 μm, 20.95 ± 9.30 mm Hg at 800 μm, Kruskal–Wallis test, *χ*^2^(3, 147) = 20.9910, *p* < 0.0001, see [20] for details). We next asked if the 1/f-like dynamics of tissue oxygenation differed between FC and FL/HL, as different power-law exponent has been observed in different brain networks [68]. We did not observe a significant difference between the fitted exponents for FC (*n =* 14 mice, 1.44 ± 0.15, average across all cortical depths for each animal) and those of the FL/HL (*n =* 23 mice, 1.41 ± 0.21, average across all cortical depths for each animal, two-sample *t* test, t(35) = 0.3710, *p* = 0.7129). We then asked if the power law fit was affected by behavior, so we fitted the power spectrum of the whole dataset including both rest and locomotion data. Including all the data increased overall power, with most of the power increase occurred at lower frequency (**Fig 1E**). Including the locomotion periods increased the power law exponent (*n =* 37 mice, rest: 1.42 ± 0.19, periods including both rest and locomotion: 1.75 ± 0.14, **Fig 1F** and **1H**, Wilcoxon signed rank test, *p* < 0.0001) but does not change the laminar differences (1.72 ± 0.23 at 100 μm, 1.77 ± 0.23 at 300 μm, 1.75 ± 0.24 at 500 μm, 1.77 ± 0.21 at 800 μm, *n* = 37 mice, **Fig 1F**, one-way ANOVA, F(3,147) = 0.3555, *p* = 0.7852), as observed using only resting data.

We further tested the existence of 1/f-like dynamics of brain oxygenation using DFA [115], which operates in the time domain. DFA measures the amount of fluctuation, F(n), of a detrended integrated signal at different length scales n, revealing the scaling properties of the signal. We fitted the fluctuations with the function F(n)∝*n*^*α*^. The parameter *α*, known as the scaling exponent, quantifies the temporal correlation in the signal as follows [126,127]: if *α* = 0.5, there is no correlation in the fluctuations, and the signal is “white noise” (**S1 Fig**, panel A); if *α* is appreciably greater than 0.5, this means there are positive correlations in the signal, where large values are more likely to be followed by large values (and vice versa; **S1 Fig**, panels C and D), which is a hallmark of 1/f-like dynamics; if *α*<0.5, there are negative correlations, where large values are more likely to be followed by small values, and vice versa. Fitting the DFA with the function F(n)∝*n*^*α*^ showed a very high goodness of fit (rest: R^2^ = 0.96 ± 0.03; all data: R^2^ = 0.98 ± 0.02; **S2 Table**), and the majority of DFA scaling exponents were greater than one (**Fig 1G**), consistent with the signal having 1/f-like dynamics. No significant differences were observed among different cortical depths using both rest and locomotion data (1.28 ± 0.15 at 100 μm, 1.34 ± 0.13 at 300 μm, 1.31 ± 0.13 at 500 μm, 1.35 ± 0.11 at 800 μm, *n =* 37 mice, **Fig 1G**, Kruskal–Wallis test, *χ*^2^(3, 147) = 4.5401, *p* = 0.2087). However, the DFA scaling exponent at rest was smaller for surface layer compared to deep layers (1.15 ± 0.15 at 100 μm, 1.25 ± 0.17 at 300 μm, 1.22 ± 0.13 at 500 μm, 1.30 ± 0.14 at 800 μm, *n* = 37 mice, **Fig 1G**, Kruskal–Wallis test, *χ*^2^(3, 143) = 13.5451, *p* = 0.0036). The DFA of the oxygen dynamics in the cortex, like the analysis in the frequency domain (**Fig 1E** and **1F**), is consistent with the oxygen dynamics having 1/f-like dynamics.

Using 2 different analysis approaches, we found that, just as in primates [8], there are 1/f-like dynamics in the oxygen levels in the cortex of mice. There was also a band-limited component (which deviates from a strict 1/f relationship), albeit at a slightly higher frequency than that found in primates [8], close to the vasomotion frequency of rodents [30].

### Fluctuations in band-limited power of the LFPs do not show 1/f-like dynamics

**Fig 2:**
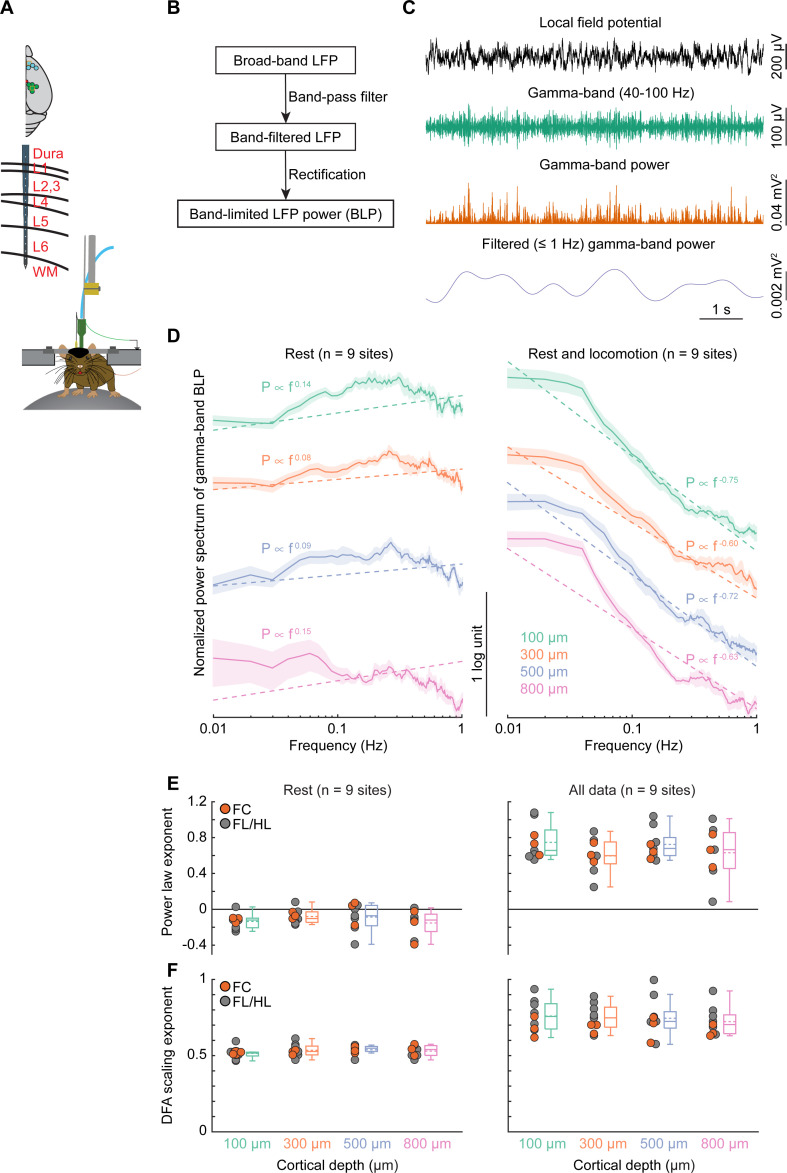
1/f-like dynamics of BLP signals for gamma-band. (**A**) Experimental setup. Top, schematic showing all laminar electrophysiology measurement sites in FC (*n =* 4 sites) and FL/HL (*n* = 6 sites). Bottom, schematic showing the layout of the electrodes and measurement depth. (**B**) Schematic outline the general method for extracting the BLP signals. Raw LFP data were first bandpass filtered and then rectified. The resulting signal was then low-pass filtered and resampled. This procedure was applied to each frequency band of the LFP. (**C**) Example traces showing the application of this scheme to one LFP signal, for the gamma-band frequency range. (**D**) Normalized (by total power between 0.01–1 Hz) power spectrum of gamma-band LFP power across cortical depth during rest (left) and periods including rest and locomotion (right). The dashed lines denote the group average linear regression fit. Data are shown as mean ± SEM. For better visualization and comparison between these signals, the power spectrum curves have been vertically shifted between different cortical depths. (**E**) Group average of power law exponent across different cortical layers during periods of rest (left, *n* = 9 sites) and periods including both rest and locomotion (right, *n* = 9 sites). Gray circles denote the measurements in FL/HL, while the orange circles denote the measurements in FC. (**F**) As (**E**) but for DFA scaling exponents. Data in (**E**) and (**F**) are shown as median ± interquartile range using boxplot, with the sample mean shown as dashed lines. The data used to generate this figure are available at https://doi.org/10.5061/dryad.pg4f4qrmt. BLP, band-limited power; DFA, detrended fluctuation analysis; FC, frontal cortex; FL/HL, forelimb/hindlimb; LFP, local field potential.

To determine whether neural activity exhibited similar dynamics to oxygen signals, we recorded LFPs (0.1 to 300 Hz) and multiunit activity (MUA; 300 to 3,000 Hz) from 16-channel laminar electrodes placed in the FC (*n =* 4 sites) and FL/HL (*n* = 6 sites) during wakeful rest and locomotion in a separate group of mice (*n* = 7 mice; **Fig 2A**). Recording from one site from FC was excluded from this analysis as there was not enough resting data (see Methods). Broadband (1 to 100 Hz) LFPs showed 1/f-like power spectra above 5 Hz, but not below, and MUA activity has a relatively smaller slope (**S2 Fig**). The most relevant aspect of the LFP for the oxygen signal is the BLP (**Fig 2B** and **2C**), which is a measure of envelope amplitude changes of LFP oscillations at specific frequency bands. Previous studies have shown that BLPs in the gamma-band (40 to 100 Hz) are best correlated with the time course of vessel dilation and oxygen changes [20,25,30–32,34,38,128], so we next calculated the power of gamma-band LFP oscillations (see Methods; **Fig 2D**) and estimated the power law fitting exponent (**Fig 2E**). In contrast to the broadband LFPs (**S2 Fig**), a flat power spectrum was observed in the gamma-band BLP in the frequency range below 1 Hz at rest (−0.14 ± 0.08 at 100 μm, −0.08 ± 0.08 at 300 μm, −0.09 ± 0.15 at 500 μm, −0.15 ± 0.15 at 800 μm, **Fig 2E**, left, one-way ANOVA, F(3,34) = 0.7794, *p* = 0.5145), characteristic of white noise (**S1 Fig**, panel A). Fitting all the data, including both rest and locomotion periods, significantly increased the power law exponent (rest: −0.11 ± 0.08, all data: 0.68 ± 0.18, Wilcoxon rank sum test, *p* < 0.0001) and showed no laminar difference (0.75 ± 0.20 at 100 μm, 0.60 ± 0.19 at 300 μm, 0.72 ± 0.17 at 500 μm, 0.63 ± 0.30 at 800 μm, one-way ANOVA, F(3,34) = 0.9981, *p* = 0.4067; **Fig 2E**, right). The power spectra of the sub-alpha (1 to 8 Hz) BLP and beta (10 to 30 Hz) BLP were also similar to white noise (**S3 Fig**). Further analysis of DFA scaling exponent reproduced all the results obtained using the power law exponent. Specifically, the DFA scaling exponent of gamma-band BLP shows characteristics of white noise at rest (0.52 ± 0.04 at 100 μm, 0.54 ± 0.04 at 300 μm, 0.54 ± 0.03 at 500 μm, 0.53 ± 0.04 at 800 μm, one-way ANOVA, F(3,35) = 0.7474, *p* = 0.5319; **Fig 2F**, left). A significant larger DFA scaling exponent was observed using both rest and locomotion periods (rest: 0.53 ± 0.03, all data: 0.74 ± 0.09, Wilcoxon rank sum test, *p* < 0.0001) and showed no laminar difference (0.76 ± 0.10 at 100 μm, 0.75 ± 0.09 at 300 μm, 0.75 ± 0.13 at 500 μm, 0.72 ± 0.09 at 800 μm, one-way ANOVA, F(3,35) = 0.1844, *p* = 0.9063; **Fig 2F**, right). This shows that the power spectra of BLP fluctuations of LFPs in mice do not have 1/f-like dynamics, consistent with recordings from nonhuman primates and humans [74,75,129].

### Weak correlations between tissue oxygenation dynamics and electrophysiology

**Fig 3:**
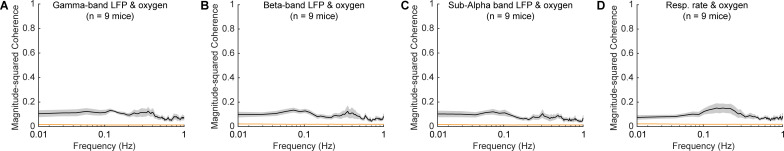
Weak coherence between BLP and tissue oxygenation. Magnitude-squared coherence between brain tissue oxygenation and gamma-band LFP power (**A**), beta-band LFP power (**B**), sub-alpha band LFP power (**C**), and respiratory rate (**D**) during rest. The orange line denotes the 95% CI of the coherence. Data are shown as mean ± SEM in (**A**−**D**). The data used to generate this figure are available at https://doi.org/10.5061/dryad.pg4f4qrmt. BLP, band-limited power; CI, confidence interval; LFP, local field potential.

The large mismatch between the power law fit exponents of the power spectrums for BLP of LFPs and oxygen fluctuations suggest that their relationship is weak. We then asked how correlated/coherent our oxygen signals were with simultaneously recorded neural activity at rest. To answer this question, we simultaneously measured tissue oxygenation, respiration, and LFP activity in 9 animals. To differentiate the frequency dependency of the correlation, we calculated the magnitude-squared coherence between oxygen and BLP of LFP, as well as the coherence between oxygen and respiratory rate (see Methods). The magnitude-squared coherence at a given frequency is equivalent to the R^2^ between the 2 signals bandpass filtered at the frequency [36]. A weak but statistically significant level of coherence between BLP of LFP and oxygen was observed between 0.01 and 1 Hz, with larger coherence at the lower frequencies (**Fig 3A**–**3C**). However, the magnitude of this squared coherence (which will report the fraction of variance in the signal at that frequency) was low, less than 0.2, implying >80% of the observed variance was not (linearly) predicted by neural activity. This low value should be viewed in light of previous work comparing the correlations of the BOLD signal with simultaneously measured electrophysiological signals in awake primates. These studies found that correlation coefficients (R) between gamma-band LFP power and the hemodynamic signals were in the range of 0.3 to 0.4 [34,128,130]. These “resting-state” correlations were consistent across a wide range of data acquisition parameters (0.25 Hz to 1 Hz sample rate) and durations (30 seconds to 30 minutes), indicating that these correlations were not dependent on acquisition parameters in this range. However, when a very strong visual stimulus and long (10 seconds) repetition time is used, the correlation coefficient can be as high as 0.8 [128]. A more recent study in mouse somatosensory cortex that controlled for arousal level also showed similar correlations between simultaneous hemodynamic and neural signals [131], suggesting that the somatosensory cortex of rodents shows similar neurovascular coupling behavior as seen in nonhuman primates [34,128,130]. The amount of variance explained (R^2^) by neural activity can be obtained by squaring the correlation coefficient, giving a value in the range of 10% to 20%, meaning that 80% to 90% of the observed BOLD signal is uncorrelated with neural activity, similar to our results.

**Fig 4:**
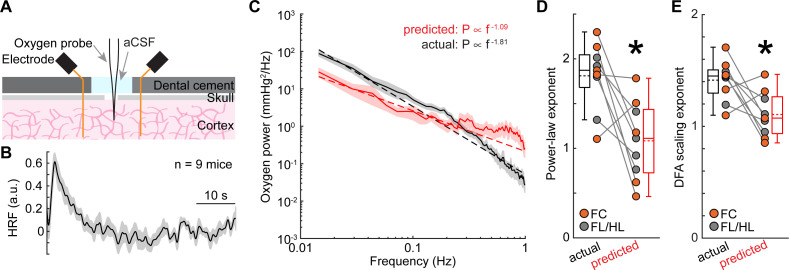
Nonneuronal factors contribute to the 1/f-like dynamics of tissue oxygenation. (**A**) Experimental setup. (**B**) Group average (*n* = 9 mice) of the HRF obtained by deconvolving brain tissue oxygenation by gamma-band power of the LFP. (**C**) Power spectrum of actual (black) and predicted (red) oxygenation. (**D**) Power law exponent of actual (black) and predicted (red) oxygenation in FL/HL (4 mice, gray circle) and FC (5 mice, orange circle). *paired *t* test, t(8) = 3.4059, *p* = 0.0093. (**E**) As (**D**) but for scaling exponent from DFA. *paired *t* test, t(8) = 2.8085, *p* = 0.0229. Data are shown as mean ± SEM in (**B**) and (**C**). Data in (**D**) and (**E**) are shown as median ± interquartile range using boxplot, with the sample mean shown as dashed lines. The data used to generate this figure are available at https://doi.org/10.5061/dryad.pg4f4qrmt. aCSF, artificial cerebrospinal fluid; DFA, detrended fluctuation analysis; FC, frontal cortex; FL/HL, forelimb/hindlimb; HRF, hemodynamic response function; LFP, local field potential.

As another test of how well the neural activity can predict changes in oxygenation, we calculated the oxygen hemodynamic response function (HRF; **Fig 4B**) by deconvolving oxygen signals from gamma-band power fluctuations of LFP [20,30], using the first half of the data from each site. We fit the deconvolved HRF with the sum of 2 gamma distribution functions (see Methods), which is standard in the field [25,37,132–135] to create a smoothed HRF. There was a good agreement between the deconvolved and smoothed HRFs (goodness of fit: 0.73 ± 0.39, median ± interquartile range). As increases in gamma-band power will lead to vasodilation and increases in oxygenation, we quantified the positive peak of the gamma distribution fitting. The positive peak of the smoothed HRF (time to peak = 2.99 ± 0.31 second, mean ± SEM, *n =* 9 mice) and the full-width at half maximum of the HRFs (3.90 ± 3.35 seconds, median ± interquartile range, *n* = 9 mice) were comparable to the dynamics of those seen in previous measurements of cerebral oxygen dynamics [97,136] and BOLD fMRI [137,138].

We then tested how well the HRF predicted tissue oxygenation from neural data. We convolved the HRF with gamma-band power fluctuations using the second half of the data, to get a simulated oxygen signal, which reflects the oxygen component predicted by neural activity (**S4 Fig**). This model uses the same assumptions built into the analysis of BOLD fMRI data, that the observed signal (oxygen concentration or BOLD) is a linear convolution of the neural activity with an HRF [20,25,30,37,128]. We then compared the power spectrum between the observed versus the predicted oxygenation, using data during periods including both rest and locomotion. We found that the oxygen concentration predicted from the neural activity only predicted a small amount of the variance (R^2^) of the signal (R^2^ = 0.04 ± 0.06, *n =* 9 mice). Furthermore, the power spectrum of the oxygen fluctuations predicted from the neural activity did not show the same frequency dependence as the actual oxygen fluctuations (**Fig 4C** and **4D**; observed: 1.81 ± 0.38, predicted: 1.09 ± 0.44, paired *t* test, t(8) = 3.4059, *p* = 0.0093). Further analysis using DFA reproduced these results (**Fig 4E**; observed: 1.41 ± 0.18, predicted: 1.11 ± 0.20, paired *t* test, t(8) = 2.8085, *p* = 0.0229). Note that the predicted power spectrum was approximately 62% smaller than the actual power spectrum at frequencies below 0.1 Hz (**Fig 4C**; paired *t* test, t(8) = 3.8597, *p* = 0.0048), indicating that putative nonneuronal components contribute more to the frequencies below 0.1 Hz. These results are consistent with the hypothesis that neural activity is not the dominant factor driving the 1/f-like dynamics in tissue oxygenation. A fundamental assumption of both our experiments and the fMRI field is that the HRF is stable over the course of minutes to hours. If the HRF changes substantially over the course of tens of minutes, any hemodynamic signals from fMRI are uninterpretable [139,140]. Consistent with this assumption, experimental measurements of HRFs in awake mice have shown that they are stable over days and are not changed by behavioral state and dynamics [30]. However, as there could be a heretofore unknown nonlinear relationship between neural activity and oxygenation, we sought to probe this relationship by suppressing local neural activity.

### Impact of suppressing neural activity on tissue oxygenation 1/f-like dynamics

**Fig 5:**
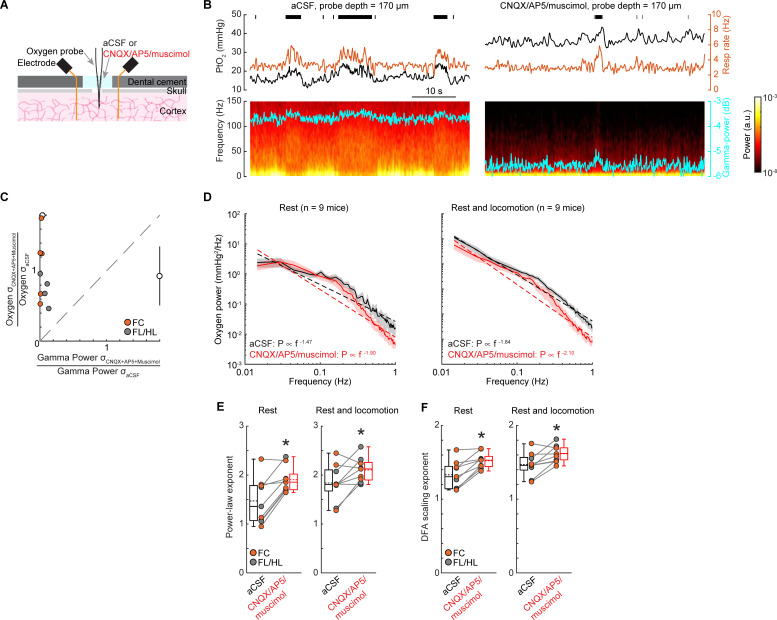
1/f-like fluctuations persist when neural activity is suppressed. (**A**) Experimental setup. (**B**) Example traces showing PtO_2_ responses to locomotion at sites 170 μm below brain surface before (left) and after (right) application of CNQX/AP5/muscimol in the well over the craniotomy. Top, black tick marks denote locomotion events; middle, PtO_2_ (black) and respiratory rate (orange) responses to locomotion; bottom, example of data showing spectrogram of LFP (cyan trace showing the gamma-band power). (**C**) Comparison of resting gamma-band power of LFP and PtO_2_ fluctuations quantified with the SD of the signal. Black circles and bars outside the axes show the population mean and SD. The dashed gray line is the unity line. Clustering of the points in the upper left corner shows a pronounced decrease in the neural activity was not accompanied by a decrease in the amplitude of the oxygen fluctuations. (**D**) Power spectrum of tissue oxygen signal before (black) and after (red) application of CNQX/AP5/muscimol using resting data (left) and data including both rest and locomotion (right). The dashed line indicates the linear regression fit. Data are shown as mean ± SEM. (**E**) Power law exponent of tissue oxygen signal before (black) and after (red) application of CNQX/AP5/muscimol using resting data (left) and data including both rest and locomotion (right). *paired *t* test, t(8) = 4.4711, *p* = 0.0021 (rest, *n =* 9 mice); t(8) = 2.5967, *p* = 0.0318 (rest and locomotion, *n* = 9 mice). (**F**) As (**E**) but for DFA scaling exponents. *paired *t* test, t(8) = 5.0287, *p* = 0.0010 (rest, *n* = 9 mice); t(8) = 2.9959, *p* = 0.0172 (rest and locomotion, *n* = 9 mice). Data in (**E**) and (**F**) are shown as median ± interquartile range using boxplot, with the sample mean shown as dashed lines. The data used to generate this figure are available at https://doi.org/10.5061/dryad.pg4f4qrmt. aCSF, artificial cerebrospinal fluid; fluid; AP5, (2R)-amino-5-phosphonopentanoic acid; CNQX, 6-cyano-7-nitroquinoxaline-2,3-dione; DFA, detrended fluctuation analysis; FC, frontal cortex; FL/HL, forelimb/hindlimb; LFP, local field potential; SD, standard deviation.

While we found that the majority of the observed oxygen fluctuations could not be explained by neural activity (**Figs 3** and **4**), if the relationship between neural activity and oxygenation is not captured by the HRF, such as the nonlinearity of brain hemodynamics [141], then some aspect of neural activity might still explain the oxygen fluctuations. To test this possibility mechanistically, we pharmacologically silenced neural activity in the cortex [20,30], which will also block increases in blood flow mediated by these increases in neural activity [20,30]. If the 1/f-like power spectrum in oxygen concentration goes away when neural activity is silenced, this would suggest that the fluctuations are due to neural activity and the subsequent vasodilation. If, however, the 1/f-like power spectrum is still present, this would suggest that these oscillations have a nonneuronal origin. To mechanistically understand whether the observed 1/f-like properties of oxygen signals is due to coherent neural activity fluctuations, we recorded tissue oxygenation and LFP simultaneously (**Fig 5A**, **S5 Fig**). Application of 6-cyano-7-nitroquinoxaline-2,3-dione (CNQX)/(2R)-amino-5-phosphonopentanoic acid (AP5)/muscimol significantly and substantially suppressed the gamma-band LFP power by 89% ± 8% (Wilcoxon signed-rank test, *p* = 0.0039) and variance by 77% ± 21% (paired *t* test, t(8) = 5.0246, *p* = 0.0010), but did not change the variance of the tissue oxygenation signal (**Fig 5B** and **5C**; paired *t* test, t(8) = 0.7542, *p* = 0.4723), which suggests that the magnitude of the brain tissue oxygenation fluctuations were not reduced by silencing neural activity.

We then asked if the suppression of neural activity alters the 1/f-like characteristics of the PtO_2_ power spectrum. If the oxygenation fluctuations are driven by neural activity, decreasing neural activity should reduce the amplitude of the oxygen fluctuations. The power spectrum of spontaneous oxygen fluctuations under artificial cerebrospinal fluid (aCSF) had a power law exponent of 1.47 ± 0.47 (*n* = 9 mice) during rest. Application of CNQX/AP5/muscimol significantly increased the power law exponent to 1.90 ± 0.27 (paired *t* test, t(8) = 4.4711, *p* = 0.0021; **Fig 5D** and **5E**) during rest. A significant increase of power law exponent was also observed when using the entire dataset (aCSF: 1.84 ± 0.38; CNQX/AP5/muscimol: 2.10 ± 0.25; *n* = 9 mice, paired *t* test, t(8) = 2.5967, *p* = 0.0318; **Fig 5D** and **5E**). The DFA scaling exponents reported similar increases using data during rest (aCSF: 1.34 ± 0.19; CNQX/AP5/muscimol: 1.53 ± 0.11; *n =* 9 mice, paired *t* test, t(8) = 5.0287, *p* = 0.0010; **Fig 5F**), as well as using the entire dataset (aCSF: 1.43 ± 0.17; CNQX/AP5/muscimol: 1.55 ± 0.11; *n =* 9 mice, paired *t* test, t(8) = 2.9959, *p* = 0.0172; **Fig 5F**). Silencing neural activity did not affect the amplitude of oxygen fluctuations below 0.1 Hz (aCSF: 7.04 ± 5.59 mm Hg^2^/Hz; CNQX/AP5/muscimol: 7.47 ± 6.60 mm Hg^2^/Hz; Wilcoxon signed-rank test, *p* = 0.8633), though there was a slight but not significant decrease in the amplitude of oxygen fluctuations above 0.1 Hz during rest (aCSF: 0.59 ± 0.31 mm Hg^2^/Hz; CNQX/AP5/muscimol: 0.37 ± 0.35 mm Hg^2^/Hz; Wilcoxon signed-rank test, *p* = 0.1359). These results reflect that the infraslow (<0.1 Hz) oscillations in brain oxygenation are not predicted by neural activity, which is consistent with the observation that these oscillations are primarily not driven by neural activity (**Fig 4C**). Taken together, these results show that suppressing neural activity did not abolish 1/f-like oscillations in tissue oxygenation or decrease the amplitude of the oxygen fluctuations, suggesting that nonneuronal contributions are a major driver of these dynamics. Notably, suppressing neural activity does not change the 1/f-like dynamics in both broadband LFP and BLP fluctuations (**S5 Fig**).

Respiration is a major factor affecting brain oxygenation [20], and fluctuations in respiration rate are known to drive substantial changes in BOLD fMRI signals [13,15,19,142]. If the respiration rate shows 1/f-like dynamics [143], this could account for the fluctuations in oxygenation that we see in the tissue when neural activity was suppressed. We found no evidence for 1/f-like dynamics in the respiration rate during rest (**S6 Fig**). Suppressing neural activity did not affect the respiration dynamics during rest (*n =* 9 mice; fitted exponent for aCSF: 0.25 ± 0.11; CNQX/AP5/muscimol: 0.21 ± 0.13; paired *t* test, t(8) = 1.5758, *p* = 0.1537). When estimated using data including both resting and locomotion, suppressing neural activity slightly reduced the power law exponent (*n* = 9 mice; aCSF: 0.60 ± 0.19; CNQX/AP5/muscimol: 0.46 ± 0.21; Wilcoxon signed-rank test, *p* = 0.1359), partially due to the fact that suppressing neural activity reduced the time mice spend locomoting. The DFA scaling exponent results were consistent with those of the power spectrum exponent (**S6 Fig**). Specifically, after suppressing neural activity, DFA scaling exponent of respiratory rate did not change using data during rest (aCSF: 0.78 ± 0.04; CNQX/AP5/muscimol: 0.77 ± 0.05; *n* = 9 mice, paired *t* test, t(8) = 0.6583, *p* = 0.5288) but reduced when it is estimated using the entire dataset (aCSF: 0.86 ± 0.06; CNQX/AP5/muscimol: 0.81 ± 0.06; *n* = 9 mice, paired *t* test, t(8) = 3.5835, *p* = 0.0072). The lack of 1/f-like dynamics in respiration signals indicates that fluctuations in blood oxygenation due to the fluctuations in respiration are not the origin of the observed 1/f-like oxygen dynamics in the cortex.

### Role of RBCs spacing variations in generating 1/f-like tissue oxygenation fluctuations

**Fig 6:**
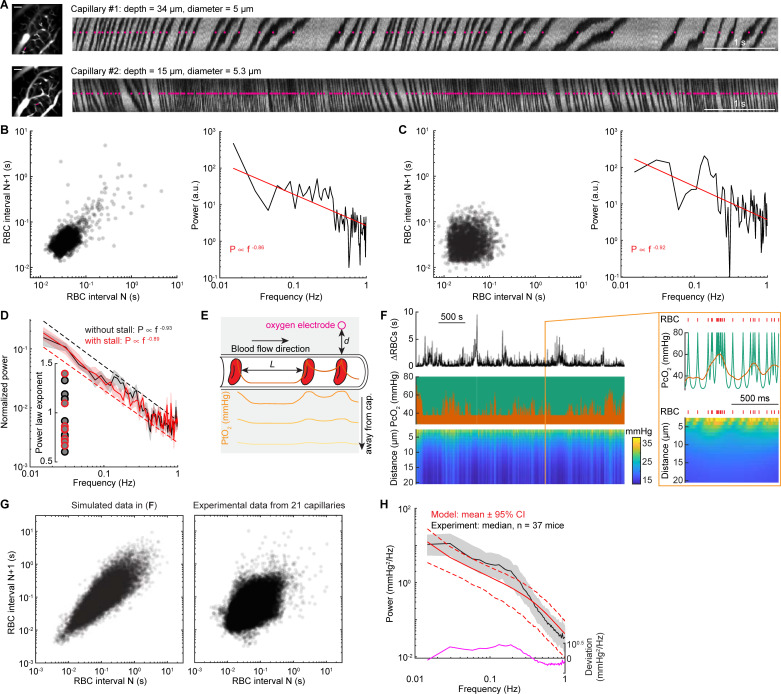
RBCs spacing heterogeneity contributes to 1/f-like oxygen fluctuations. (**A**) Representative line scan images showing the RBCs spacing in 2 different capillaries with (top) and without (bottom) stall events in an example mouse during rest. The images on the left showing the vasculature around the measurement sites, as indicated by the magenta line. Scale bar = 50 μm. The magenta dots indicate the detected RBCs. (**B**) Left, Poincare plot showing the relationship between consecutive RBC spacing intervals (i.e., autocorrelation trend) of capillary #1. The dark area indicates the density of the RBC spacing. Right, power spectrum for the RBC spacing of capillary #1. The Poincare plot showing 3,341 RBCs in a 142.8-second resting period. (**C**) As (**B**) but for capillary #2. The Poincare plot showing 2,603 RBCs in a 100.4-second resting period. (**D**) Group average of power spectrum of inter-RBC transit time for data without stall events (black, *n =* 13 capillaries) and with stall events (red, *n* = 8 capillaries). Inset, fitted power law exponent for each of the capillaries with (red circle) and without (black circle) stall events. (**E**) Schematic showing that PO_2_ measured at the RBC border decreases with distance and reaches its lowest value between 2 RBCs. Orange line inside the capillary denotes the oxygenation carried by the RBC and the plasma. The gray shaded area denotes brain tissue. Solid traces inside the gray shaded area denote PtO_2_ at different distance away from the capillary wall (as indicated by the arrow). (**F**) Simulated data example showing the fluctuations of RBC spacing (top), oxygenation in the capillary (middle, green), and oxygen in the tissue (bottom) generated by simulating a Krogh cylinder of 20 μm radius supplied by a capillary of 3 μm radius. The orange trace (middle) denotes the simulated tissue oxygenation fluctuations close to the capillary wall, counting in the low-pass filtering nature of the oxygen diffusion dynamics and the response properties of the polarographic electrode. The orange box denotes a 1-second segment of the dataset. The red tick marks denote the passage of a single RBC. (**G**) Poincare plot showing the relationship between consecutive RBC spacing intervals for the simulated data example (left) shown in (**F**) and all experimental data from 21 capillaries (right). (**H**) Comparison of power spectrum of tissue oxygenation measured using polarographic electrodes (black) and generated using the simple model (red). The black trace denotes the median of all power spectrum from experiments. The edge of the gray shaded area indicates the 75 percentiles and 25 percentiles of the experimental data. The red line denotes the mean (solid line) and the 95% CI (dashed line) from the model. The magenta line denotes the deviation between the experimental and simulated data. The data used to generate this figure are available at https://doi.org/10.5061/dryad.pg4f4qrmt. CI, confidence interval; RBC, red blood cell.

The vast majority of oxygen in the blood is carried by RBCs, with the plasma carrying a small fraction of the total oxygen [88,93,95,96], which means that heterogeneities in RBCs densities will cause changes in local oxygen supply [77,80,82,88,93–96]. It has long been appreciated from theoretical models that the tissue oxygenation can vary with the passage of a single RBC, creating an EAT in tissue oxygenation [76–92]. Recent high-resolution measurements of oxygenation with phosphorescent dyes have confirmed the existence of these transients [77,82,93–96], but these measurements require aligning the signals to the passage of the RBCs and would not be able to assay any slow oxygenation change that drive 1/f-like dynamics. Notably, oxygen-sensitive electrodes used in the present study lack the temporal resolution to detect individual EATs. As RBCs transit through the capillaries in single file flow, the tissue oxygenation outside the capillary fluctuates with their density (**Fig 6E**). Interestingly, there are infrequent “stalls” in RBCs flow in capillaries, caused by leukocytes transiently blocking flow [98–100,103,144,145]. During a stall, a large RBC spacing will result in a sudden drop of tissue oxygenation within approximately 15 μm [123] of the capillary. As theoretical work has shown that pulsatile time series generate 1/f-like spectra [146], we asked if the variations in RBCs density through single capillaries had 1/f-like dynamics.

To answer this question, we measured inter-RBCs spacing in capillaries using 2PLSM to perform line scans along individual capillaries [147]. The plasma was labeled with a fluorescent dye, and RBCs appear as dark streaks (**Fig 6A**). The pattern of RBCs and plasma was thresholded and binarized to generate a train of point processes (**Fig 6A**). A “stall” event was defined as an inter-RBC spacing greater than 1 second [103]. For the following power spectrum analysis and modeling, we only used RBCs spacing intervals during long resting segments (i.e., ≥60 seconds; see Methods). For all the RBC intervals during long resting periods (approximately 66 minutes data from 21 capillaries in 9 mice; 22.4 ± 30.7 ms, median ± interquartile range; 95% confidence interval: [5.1 ms, 159.4 ms]), only approximately 0.06% RBC intervals are stall events (1.65 ± 1.32 second, median ± interquartile range; 95% confidence interval: [1.01 second, 13.11 seconds]; **Fig 6G**). This rare occurrence of “stall” events is consistent with previous work conducted in awake rodents [102,144] but smaller than anesthetized rodents [102,103,105,144]. In addition, the consecutive RBC intervals are correlated, i.e., a long RBCs interval tends to be followed by another long RBCs interval, and vice versa (**Fig 6B** and **6C**). We first quantified the nature of the power spectrum of RBC arrival fluctuations during rest by fitting the power spectrum of the binarized data (0: plasma; 1: RBC) with a power law distribution in the 0.01 to 1 Hz frequency range. Binarization makes sense, as the oxygen levels will be high as an RBC passes by, and low when there is only plasma present. We observed 1/f-like dynamics of RBCs spacing, with the exponent range from 0.6 to 1.4 (0.91 ± 0.23, 21 capillaries in 9 mice, **Fig 6B**–**6D**). Note that the rare occurrence of RBCs transient “stall” events did not significantly affect the fitted exponent (**Fig 6D**; 0.89 ± 0.24, 8 capillaries with stall events; 0.93 ± 0.23, 13 capillaries without stall events; two-sample *t* test, t(19) = 0.3558, *p* = 0.7259).

We then developed a simple computational model (see Methods) to determine how the delivery of oxygen by RBCs is affected by the statistics of RBC passages. Given the volume of tissue sampled by the electrode [148], and the spacing of capillaries [123], the oxygen signal at our electrode will be dominated by the nearest capillary. We generated a time series of RBCs spacing utilizing data from our 2PLSM observations and data from previous studies [102,144]. We only considered the tissue oxygenation transients caused by the RBCs spacing. The RBCs (with high PO_2_) and plasma gaps (with relatively low PO_2_) alternately passed through the capillary, and the EAT can be visualized by considering a fixed measurement site on the capillary wall (**Fig 6E**). As the tissue response time is much slower compared to the RBC transit time (due to the low-pass filtering nature of the oxygen diffusion dynamics), the oxygen delivery from capillaries decays rapidly with distance at higher temporal frequencies. This means that the tissue oxygenation will be a smoothed version of the EATs (**Fig 6E**). In the model, the RBCs were considered as a point process, and the EATs surrounding each RBC were modeled using an exponential decay (**Fig 6E**), based on the data measured using two-photon phosphorescent imaging [93–96]. The tissue response time [83] was also considered when calculating the tissue oxygen at different distances from the capillary wall, as oxygen delivery from capillaries decays rapidly with distance at high frequencies of pulsatile flow in the vessels (**Fig 6E**).

We generated simulations of oxygenation of comparable durations as our experiments using polarographic electrodes (approximately 40 minutes) and examined the power spectrum. **Fig 6F** illustrated a representative RBC train we modeled with 0.5% [102,144] stall events and a power spectrum with a fitted exponent 1. For this specific example, based on measurements in awake animals [95,96], we assumed that oxygenation of each RBC (PO_2_ = 80 mm Hg) and oxygenation in each plasma gap (PO_2_interRBC = 28 mm Hg) were constant, giving an EAT magnitude of 52 mm Hg. The generated data have shown that RBC spacing fluctuations have long-range autocorrelations (**Fig 6G**), as seen in our experimental data (**Fig 6B**, **6C** and **6G**). To further quantitatively validate this model, we generated 1,000 RBC time series, each with a randomly assigned power law exponent based on our experimentally determined range (**Fig 6D**). We then randomly assigned each RBC time series a flow rate, which will determine the EATs, based on previous studies [95,96], to simulate brain tissue oxygenation dynamics. The simulations using the simple model showed very close agreement with our experimental data (**Fig 6H**). Specifically, approximately 70% of the experimentally observed power law exponent are covered by our model (95% confidence interval: [0.93, 1.60]). However, our model slightly underestimated the fluctuations below 0.2 Hz, as indicated by the deviation between the power spectrum of the model and our experiments (**Fig 6H**). This partially contributes to the smaller power law exponent reported by our model (model: 1.24 ± 0.21, experimental data: 1.42 ± 0.19, Wilcoxon rank sum test, *p* < 0.0001). The underestimate of power at approximately 0.2 Hz suggests that other slow or ultraslow processes [149], such as vasomotion [30], cortical state changes, or crosstalk between the vasculature network [150,151], contributing to the 1/f-like dynamics of brain tissue oxygenation (see Discussion).

Taken together, our simulations show that variations of RBC spacing alone can generate 1/f-like oxygen fluctuations in the capillaries and surrounding tissue. This simple model points to an intriguing possibility that the discrete nature of RBCs may play an important role in 1/f-like dynamics of the oxygen levels. As the fluctuations of RBCs are largely attributed to nonneuronal mechanisms [145,152], this suggests a nonneuronal origin of 1/f-like oscillations in tissue oxygenation.

## Discussion

We found that oxygen dynamics in the mouse cortex show large, low-frequency oscillations, which were small or absent in the BLP of the LFP. These fluctuations were present in all cortical layers and multiple regions both when the mouse was at rest and during behavior. These fluctuations were weakly correlated with neural activity and persisted when neural activity was pharmacologically suppressed. Simulations based on physiological measurements showed that the stochastic, correlated fluctuations in the number of RBCs could account for driving these dynamics.

Our experimental observations and modeling have shown that RBCs spacing heterogeneity could be a contributing factor to the 1/f-like dynamics of the brain tissue oxygenation. Using only the temporal heterogeneity of RBCs, our model can generate the 1/f-like dynamics of oxygenation in the capillary and nearby tissue that it supplies with oxygen (**Fig 6**). This simple model also explains our observations of increased brain tissue oxygen power law exponent after CNQX/AP5/muscimol infusion (**Fig 5**), as the suppression of neural activity has been shown to cause arterial vasoconstriction leading to decreased flow through capillaries [44,153]. The decrease in flow will increase the probability of long stall events [144], which should significantly contribute to the brain oxygenation fluctuations at lower frequencies, as is seen in our experiments (**Fig 5**). Notably, there are a variety of factors influencing RBC heterogeneity in a single capillary. In addition to stalls in flow caused by vessel occlusion by leukocytes [98,99,103,105,144,145], the very nature of the RBC-plasma suspension will drive low-frequency fluctuations in flow and RBC heterogeneity [152,154]. RBCs have different rheological properties than the plasma [155,156], making the Newtonian descriptions of fluid flow that works for larger vessels inapplicable to the flow through capillaries. Because of the higher effective viscosity of RBC than the plasma, stochastic fluctuations in the number of RBC in a vessel changes the effective resistance of the vessel, which can lead to low-frequency fluctuations in RBC flow [152,154].

Though we modeled the RBCs and oxygenation dynamics in one single capillary, the observed oxygen dynamics may reflect a network effort from different capillaries supplying the same area [150–152,154]. In a capillary network, altered RBCs distribution due to capillary dilation/constriction has been shown to be important for the local regulation of oxygen delivery [154]. Contractions of pericytes on the time scales of tens or hundreds of seconds [157] could also regulate local RBC flows in individual capillaries. Fluctuations in flow could lead to large fluctuations of oxygen supply [105]. Simulations of RBCs heterogeneity in skeletal muscle also suggest that the capillary network is a source of spatial and temporal heterogeneity of RBC flow, and increasing number of RBCs entering the network decreases the spatial heterogeneity [158]. This more uniform RBC distribution will eliminate the slow, high-amplitude variations of oxygenation, which will explain the observation that brain oxygenation has relatively less low-frequency fluctuations at control compared to those after silencing neural activity (**Fig 5**).

### Other potential drivers of 1/f-like oxygen fluctuations

While our work suggests that stochastic fluctuations in RBCs density could be an important driver of 1/f-like fluctuations in tissue oxygenation, there are likely to be others. In addition to the neurally controlled component of hemodynamics signals, brain hemodynamics are also shown to be coupled to (and modulated by) other processes [149]. Fluctuations in hemodynamic signals can arise from slow changes in arousal state or neuromodulation. Task-associated changes in cortical state have been shown to be associated with cerebral blood volume change [159], and during sleep, there are arterial dilation-driven BOLD signal increases in the cortex [131,160]. There is also evidence that cholinergic [161], dopaminergic [162,163], and noradrenergic [129,164,165] signaling directly modulates vessel diameter. Although these neuromodulation input from subcortical nuclei can affect hemodynamic responses via modulation of neuronal responses [166], they can also affect brain blood flow through direct modulation of microvessels [167,168], via astrocytes [164,165] or perictyes [169]. Moreover, spontaneous astrocytic calcium signals can also drive arteriole diameter change, which contribute to slow oscillations of arteriole diameter (approximately 0.002 Hz in awake mice and approximately 0.0002 Hz in anesthetized mice) [170]. Calcium signals in astrocyte endfeet can also be driven by spontaneous locomotion [165,171]. Another possible driver is the ongoing vascular fluctuations, i.e., vessel autonomous oscillations in the arterial diameter, which have been found in arteries throughout the body (see [149,172] for review). The spontaneous oscillations in arterial diameters are independent of local neural activity and will additively interact with vasodilatory signals from neurons to add “noise” to the hemodynamic signal, particularly when the brain is at rest and neural activity is low [30], which will deviate the signal from pure 1/f-like [8]. Vasomotor oscillations in arteriole diameter drive oscillations in the velocity of RBCs in microvessels [100] and changes in blood oxygenation in the brain [31,173]. Critically, vasomotion is only slightly reduced in amplitude when cortical activity is silenced [30], showing that these oscillations are independent of local neural control.

As the tissue oxygenation is determined by both the supply and consumption of oxygen, fluctuations in oxygen consumption could potentially contribute to the fluctuations we observe. However, there is evidence that the neurons that control blood flow are not necessarily the most metabolically demanding ones. Optogenetic or chemogenetic stimulation of nitric oxide synthase [174] expressing interneurons drives an increase in blood flow and arterial diameter [44,175–177], minimal increase in cerebral metabolic rate of oxygen (CMRO_2_) [175], with no increase (and usually a decrease) in electrical activity [44,175]. Optogenetic or chemogenetic stimulation of pyramidal neurons drives increases in electrical activity [44,175], minimal increases in blood flow [175], and large increases in CMRO_2_ [175]. Fluctuations in the consumption of oxygen by mitochondria have 1/f-like dynamics both at the single cell level [178–181] and at a level of whole body oxygen consumption [182]. Moreover, measurements in awake and sleeping cats have shown that existence of spontaneous oscillations of cytochrome c oxidase redox state (an index of metabolism) in cortex that is not directly related to neural activity [183]. In contrast to these in vivo studies, oxygen consumption by neurons in rat hippocampal slices is closely tied to neural activity [184]. Note that oxygen levels in the slices lack the fluctuations seen in perfused tissue and the neurons are inactive. This suggests that metabolic activity that is unrelated to electrical signaling could be a potential contributor to the observed oxygen 1/f-like dynamics. Therefore, these fluctuations in oxygen consumptions should be considered in light of the brain oxygen supply. Under normal physiological conditions, the relative contribution of brain metabolism may be not that large, as the ratio between changes in blood flow and cerebral metabolism (CBF/CMRO_2_) has been estimated to be in the range of 2 to 4 (see [185] for review), which means a 10% consumption of oxygen will be accompanied by 20% to 40% increase of blood flow.

### Relating tissue oxygen signals to BOLD fMRI

How are the tissue oxygenation signals we recorded related to BOLD fMRI signals? Polarographic electrodes and BOLD fMRI sample oxygenation at different spatial and temporal scales. Polarographic electrodes have higher temporal resolution than BOLD, though there are fMRI paradigms that can image at higher resolution [134]. With our polarographic electrodes, oxygen levels are recorded from a sphere of brain parenchyma approximately 20 μm in diameter, and the temporal frequency is limited by a low-pass filter set to 1 Hz. Due to the small size of the polarographic probe, our measurements reflect oxygenation at a single capillary level, while BOLD signal primarily originates from the oxygenation of post-capillary blood vessels (e.g., venules and pial veins) [22], which will report the average of oxygenation over a larger section of tissue [136,186–188]. The BOLD signal in the veins will report the average of oxygenation of the capillaries feeding into them [189]. Because of this, the heterogeneity of RBCs in a single capillary will likely not appreciably affect blood oxygenation oscillations in the venous compartment, and, therefore, the BOLD signal. How exactly the RBCs heterogeneity caused oxygen fluctuations contribute to the 1/f-like dynamics in BOLD fMRI is still unclear. Theoretical models have shown that changes of RBCs distribution in one capillary can affect the RBCs distributions in other capillaries of the same network [152,154], which will change the vascular resistance and capillary transit time heterogeneity [190,191], and further affect the blood flow patterns and oxygen availability at the venous end [191]. Future work combing simultaneous fMRI measures and tissue oxygenation would provide a definitive answer.

While there are likely to be differences between these 2 signals, the consensus of current work is that polarography electrode measurements of oxygen in the tissue will give signals similar to those observed with BOLD fMRI, especially for stimulus-evoked responses. In nonhuman primates, polarographic signals are highly correlated with BOLD signals obtained from the same brain region [67], suggesting that the oxygen dynamics in the small volumes of tissue measured by the polarographic electrode are similar to those in the veins. Oxygen-sensitive dye measures in brain tissue also show similar dynamics to BOLD signals [192]. Finally, simulations constrained by vascular oxygenation measurements have shown that the oxygen levels in veins track capillary oxygenation very closely [136].

### Limitations

Although our results suggest that the heterogeneity of RBCs spacing contribute to the 1/f-like dynamics, there are known unknowns. How exactly the stalling events contribute to the dynamics is uncertain. Specifically, how RBC stalling in one vessel affects other vessels in the network is unclear. Is there a compensating increase of oxygen delivery in other vessels? How the interaction among vessels in the capillary network [150,151,154,193–196] affects the 1/f-like dynamics requires further investigation. In addition, one potential caveat of the study is the degree to which the activity we observed is in the pure “resting-state” regime. While it is possible to find relatively long intervals during which the mouse does not move (which we defined as rest), this is not a pure resting state, and fidgeting behavior [107–109] may trigger brain hemodynamic oscillations and contribute to 1/f-like dynamics in brain oxygenation.

### Summary

Stochastic passage of RBCs could contribute to the 1/f-like dynamics in tissue oxygenation and could potentially explain many disparate observations in the literature. It would explain why 1/f-like dynamics are seen in tissue oxygenations throughout the body, not only in the brain [1,5,8,9], why we see similar oxygen dynamics across layers and cortical regions (**Fig 1**), even though there are large differences in neural activity and vascular density across regions and layers [123–125]. Finally, fluctuations in oxygenation generated by the stochastic passage of RBCs are effectively “noise” and could explain the relatively low correlations and coherences between oxygen and neural activity observed both in our experiments and in BOLD fMRI measures [34,128,130,197]. Thus, the intermittent flow and stalling of RBCs could contribute to fluctuations in oxygenation on the time scale of seconds to minutes, as well as potentially driving neurodegenerative diseases [98,99,102,105,198,199].

## Methods

Portions of the data used in this study have been published previously [20]. This study was performed in strict accordance with the recommendations in the Guide for the Care and Use of Laboratory Animals of the National Institutes of Health. All procedures were performed in accordance with protocols approved by the Institutional Animal Care and Use Committee (IACUC) of the Pennsylvania State University (protocol #201042827).

### Animals

A total of 56 C57BL/6J mice (3 to 12 months old, 25 to 40 g, Jackson Laboratory, Bar Harbor, Maine) were used. Recordings of laminar cortical tissue oxygenation were made from 37 mice [23 (13 male and 10 female) in the somatosensory cortex (FL/HL) and 14 (7 male and 7 female) in the FC] using Clark-type polarographic microelectrode. Simultaneous measurements of cortical tissue oxygenation using polarographic electrodes, respiration, and LFP were conducted in 9 mice [5 (4 male and 1 female) in FL/HL and 4 (2 male and 2 female) in FC]. Six of these mice were also used for laminar cortical tissue oxygenation measurements. LFP and spiking activity of different cortical layers were measured using laminar electrodes in a separate set of 7 male mice (4 in FC and 6 in FL/HL, 3 mice were measured in both FL/HL and FC simultaneously). 2PLSM imaging was conducted in 9 mice (21 capillaries, 7 male and 2 female, in FL/HL). Mice were given food and water ad libitum and maintained on 12-hour (7:00 to 19:00) light/dark cycles. All experiments were conducted during the light period of the cycle.

### Surgery

All surgeries were performed under isoflurane anesthesia (in oxygen, 5% for induction and 1.5% to 2% for maintenance). A custom-machined titanium head bolt was attached to the skull with cyanoacrylate glue (#32002, Vibra-Tite, Troy, Michigan). The head bolt was positioned along the midline and just posterior to the lambda cranial suture. Two self-tapping 3/32″ #000 screws (J. I. Morris, Oxford, Massachusetts) were implanted into the skull contralateral to the measurement sites over the frontal lobe and parietal lobe. A stainless steel wire (#792800, A-M Systems, Sequim, Washington) was wrapped around the screw implanted in the frontal bone for use as an electrical ground for cortical tissue oxygenation and neural recordings. For capillary blood flow velocity measurements using 2PLSM (*n =* 9 mice), a polished and reinforced thin-skull (PoRTS) window was made covering the right hemisphere as described previously [20,24,30,59,112,200]. For simultaneous measurement of tissue oxygenation and neural activity (*n* = 9 mice), we implanted 2 electrodes to measure LFP signals differentially. Electrodes were made from Teflon-coated tungsten wire (#795500, A-M Systems) with approximately 1 mm insulation striped around the tip. The electrodes were inserted into the cortex to a depth of 800 μm at 45° angle along the rostral/caudal axis using a micromanipulator (MP-285, Sutter Instrument, Novato, California) through 2 small holes made in the skull. The 2 holes for the electrodes were made approximately 1 to 1.5 mm apart to allow insertion of the oxygen probe between the 2 electrodes in following experiments. The holes were then sealed with cyanoacrylate glue. Following the surgery, mice were then returned to their home cage for recovery for at least 1 week and then habituated to head fixation on the spherical treadmill. Habituation sessions were performed 2 to 4 times per day over the course of 1 week, with the duration increasing from 5 minutes to 45 minutes.

### Physiological measurements

Data from all experiments, except experiments using 2PLSM, were collected using custom software written in LabVIEW (version 2014, National Instruments, Austin, Texas).

#### Behavioral measurement

The treadmill movements were used to quantify the locomotion events of the mouse. The animal was also monitored using a webcam (Microsoft LifeCam Cinema, Redmond, Washington) as an additional behavioral measurement.

#### Cerebral tissue oxygenation measurement using polarographic electrode

On the day of measurement, the mouse was anesthetized with isoflurane (5% for induction and 2% for maintenance) for a short surgical procedure (approximately 20 minutes). A small (approximately 100 × 100 μm) craniotomy was made over the FC (1.0 to 3.0 mm rostral and 1.0 to 2.5 mm lateral from bregma) or the FL/HL representation in the somatosensory cortex (0.5 to 1.0 mm caudal and 1.0 to 2.5 mm lateral from bregma), and dura was carefully removed. The craniotomy was kept moist with warm aCSF and porcine gelatin (Vetspon, Greenfield, Indiana). The mouse was then moved to and head fixed upon the spherical treadmill. Oxygen measurements started at least 1 hour after cessation of anesthesia to minimize the effects of anesthesia [95,106,201].

Cerebral tissue oxygenation was recorded with a Clark-type oxygen microelectrode (OX-10, Unisense A/S, Aarhus, Denmark). A total of 9 oxygen electrodes were used in this study, with an average response time of 0.33 ± 0.11 seconds (*n* = 9 probes). No compensation for the response delay in the electrode was made. The oxygen electrodes were calibrated in air-saturated 0.9% sodium chloride (at 37°C) and oxygen-free standard solution [0.1 M sodium hydroxide (SX0607H-6, Sigma-Aldrich, St. Louis, Missouri) and 0.1 M sodium ascorbate (A7631, Sigma-Aldrich) in 0.9% sodium chloride] before and after each experiment. The linear drift of the oxygen electrode signal (1.86% ± 1.19% per hour) was corrected by linearly interpolating between pre and postexperiment calibrations. The oxygen electrode was connected to a high-impedance picoammeter (OXYMeter, Unisense A/S, Aarhus, Denmark), whose output signals were digitalized at 1,000 Hz (PCI-6259, National Instruments). Current recordings were transformed to millimeters of mercury (mm Hg) using the calibrations with air-saturated and oxygen-free solutions.

Oxygen electrodes allow long-duration, quantitative measurements of the average oxygen tension from a small volume (approximately 20 μm radius) of parenchymal tissue. The stability of the electrode provides long duration measurements, which are required to estimating the power at ultralow frequencies. For oxygen polarography measurements, the oxygen microelectrode was positioned perpendicular to the brain surface and advanced into the cortex with a micromanipulator (MP-285, Sutter Instrument). Measurement site was chosen to avoid large pial vessels. The depth zero was defined as when the tip of the oxygen microelectrode touches the brain surface under visual inspection. The probe was then advanced to depth of 100, 300, 500, and 800 μm below the pia, and 30 to 40 minutes data were recorded for each depth. After advancing the electrode, we waited at least 5 minutes before the start of each recording.

In experiments investigating effects of suppressing neural activity on cortical tissue oxygenation dynamics, a cocktail of ionotropic glutamate receptor antagonists CNQX (0.6 mM), NMDA receptor antagonist AP5 (2.5 mM), and GABA_A_ receptor agonist muscimol (10 mM) were applied to suppress neural activity. All drugs were applied topically over the craniotomy and were allowed to diffuse into the cortical tissue for at least 90 minutes before the oxygen measurements. The efficacy of the CNQX/AP5/muscimol cocktail was validated with simultaneously recorded neural activity. Neural data were amplified 1,000× and filtered (0.1 to 10k Hz, DAM80, World Precision Instruments, Sarasota, Florida) and then sampled at 30k Hz (PCI-6259, National Instruments). The oxygen signal in these experiments was recorded at a depth of approximately 100 to 200 μm.

In experiments investigating effects of suppressing neural activity on cortical tissue oxygenation dynamics, respiration was also simultaneously recorded. Measurements of breathing were taken using 40-gauge K-type thermocouples (TC-TT-K-40-36, Omega Engineering, Norwalk, Connecticut) placed near the mouse’s nose (approximately 1 mm), with care taken to not contact the whiskers. Data were amplified 2,000×, filtered below 30 Hz (Model 440, Brownlee Precision, Santa Clara, California), and sampled at 1,000 Hz (PCI-6259, National Instruments). Downward and upward deflections in respiration recordings correspond to inspiratory and expiratory phases of the respiratory cycle, respectively. We identified the time of each expiratory peak in the entire record as the zero-crossing point of the first derivative of the thermocouple signal.

At the end of the experiment, the mouse was deeply anesthetized, and a fiduciary mark was made by advancing an electrode (0.005″ stainless steel wire, catalog #794800, A-M Systems) into the brain with a micromanipulator to mark the oxygen measurement site.

#### Laminar electrophysiology

Laminar electrophysiology recordings were performed in a separate set of mice (*n =* 7). On the day of measurement, the mouse was anesthetized using isoflurane (in oxygen, 5% for induction and 2% for maintenance). Two small (1 × 1 mm^2^) craniotomies were performed over the FC (1.0 to 2.5 mm rostral and 1.0 to 2.5 mm lateral from bregma) and FL/HL representation in the somatosensory cortex (0.5 to 1.0 mm caudal and 1.0 to 2.5 mm lateral from bregma) over the contralateral hemisphere, and the dura was carefully removed. The craniotomies were then moistened with warm saline and porcine gelatin (Vetspon). After this short surgical procedure (approximately 20 minutes), the mouse was then transferred to the treadmill where it was head fixed. Measurements started at least 1 hour after the cessation of anesthesia [106,201].

Neural activity signals were recorded using 2 linear microelectrode arrays (A1x16-3mm-100-703-A16, NeuroNexus Technologies, Ann Arbor, Michigan). The electrode array consisted of a single shank with 16 individual electrodes with 100 μm interelectrode spacing. The signals were digitalized and streamed to SmartBox via a SmartLink headstage (NeuroNexus Technologies). The arrays were positioned perpendicular to the cortical surface; one was placed in the FL/HL, and the other one was placed in the FC on the contralateral side. Recording depth was inferred from manipulator (MP-285, Sutter Instrument) readings. The neural signals were filtered (0.1 to 10k Hz bandpass), sampled at 20k Hz using SmartBox 2.0 software (NeuroNexus Technologies).

#### Measuring RBC spacing in capillaries using 2PLSM

Two-photon imaging was performed with a Sutter Moveable Objective Microscope. A MaiTai HP (Spectra-Physics, Santa Clara, California) laser tuned to 800 nm was used for fluorophore excitation. Before imaging, the mouse was briefly anesthetized with isoflurane (5% in oxygen), retro-orbitally injected with 50 μL of 70 kDa fluorescein-conjugated dextran (Sigma-Aldrich) prepared at a concentration of 5% (weight/volume) in sterile saline to label plasma, and then fixed on a spherical treadmill. Imaging was done with a 20X, 1.0 NA objective (Olympus, XLUMPFLN). Control of 2PLSM and data acquisition was accomplished using MScan software (Sutter Instruments). All imaging with the water-immersion lens was done with room temperature distilled water. Wide-field images were collected to generate vascular maps of the entire window for navigational purposes. High-resolution images of the vasculature were collected using a 500 μm by 500 μm field for measurement of capillary diameter. Capillary diameter was measured using ImageJ software. To measure RBC velocity and RBC spacing, line scan images were collected from individual capillaries. RBCs appeared as tilted dark shadows on a bright background due to the fluorescein-conjugated dextran contained in the blood plasma (**Fig 6A**), and these shadows were counted.

### Data analysis

All data analyses were performed in MATLAB (R2015b, MathWorks, Natick, Massachusetts) using custom code.

#### Locomotion events identification

Locomotion events [20,24,30,202] from the spherical treadmill were identified by first applying a low-pass filter (10 Hz, fifth order Butterworth) to the velocity signal from the optical rotary encoder, and then the absolute value of acceleration (first derivative of the velocity signal) was thresholded at 3 cm/s^2^. Periods of locomotion were categorized based on the binarized detection of the treadmill acceleration:

$$\delta (t)=H(|a_{t}|-a_{c})=\left\{ \begin{array}{l} 1,\;|a_{t}|\geq a_{c}\\ 0,\;|a_{t}|<a_{c} \end{array}\right.$$

where *a*_*t*_ is the acceleration at time t, and *a*_*c*_ is the treadmill acceleration threshold.

#### Spontaneous activity

To characterize spontaneous (non-locomotion-evoked) activity, we defined “resting” periods as periods at least 4 seconds after the end of previous locomotion event and lasting no less than 60 seconds.

#### Oxygen data preprocessing

Oxygen data from polarographic electrodes were first low-pass filtered (1 Hz, fifth order Butterworth). The oxygen data were then downsampled to 30 Hz to align with binarized locomotion events.

#### Laminar neural activity

The neural signal was first digital filtered to obtain the LFP (0.1 to 300 Hz, fifth order Butterworth) and MUA (300 to 3,000 Hz, fifth order Butterworth) [20,24,30]. Time-frequency analysis of LFP signal was conducted using multitaper techniques (Chronux toolbox version 2.11, http://chronux.org/)) [114]. The power spectrum was estimated with a 1-second window with approximately 1 Hz bandwidth averaged over 9 tapers. MUA signals were low-pass filtered (5 Hz, Bessel filter). Spike rate was obtained by counting the numbers of events that exceed an amplitude threshold (3 standard deviations (SDs) above background) in each 1 ms bin.

To examine raw LFP or BLP modulations at different frequency bands, we first used a third-order Butterworth filter to apply zero-phase bandpass filtering to the raw LFP according to the following frequency bands: sub-alpha, 1 to 8 Hz; beta, 10 to 30 Hz; and gamma: 40 to 100 Hz. The resulting BLP signals were squared and full-wave rectified. They were then resampled to 20 Hz after low-pass filtering below 1 Hz. These steps are illustrated in **Fig 2B** and **2C**.

The spike train data were extracted from each channel of the laminar electrode. Firing-rate signals in these data were smoothed with a Gaussian kernel with full-width at half maximum of 10 ms to generate a continuous firing rate signal.

#### Magnitude-squared coherence

We used coherence analysis [203] to reveal correlated oscillations and deduce functional coupling among different signals. The magnitude squared ordinary coherence between 2 signals x and y are defined as

$$C_{xy}^{2}(f)=\frac{S_{xy}^{2}(f)}{S_{x}(f)S_{y}(f)},$$

where *S*_*x*_(*f*) and *S*_*y*_(*f*) are the autocorrelation spectra of the signals, and *S*_*xy*_(*f*) is the cross-correlation spectrum.

#### Quantifying the oxygen fluctuations predicted by the neural activity

We considered the neurovascular relationship to be a linear time invariant system [37,139,204]. To provide a model-free approach to assess the relationship between tissue oxygenation and neural activity, HRF was calculated by deconvoluting tissue oxygenation signal to gamma-band LFP power, using the following equation:

$$H_{(k+1)\times 1}={\left(L^{T}L\right)}^{-1}L^{T}V_{(m+k)\times 1}$$

H is the HRF, and V is the tissue oxygenation signal. L is a Toeplitz matrix of size (m + k) × (k + 1) containing measurements of gamma-band LFP power (n):

$$L(\overset{⃑}{n})=\left(\begin{array}{cccccc} 1 & n_{1} & 0 & 0 & \cdots & 0\\ 1 & n_{2} & n_{1} & 0 & \cdots & 0\\ \vdots & \vdots & n_{2} & n_{1} & \cdots & \vdots \\ \vdots & n_{k} & \vdots & n_{2} & \cdots & n_{1}\\ \vdots & 0 & n_{k} & \vdots & \cdots & n_{2}\\ \vdots & \vdots & \vdots & n_{k} & \ddots & \vdots \\ 1 & 0 & 0 & 0 & \cdots & n_{k} \end{array}\right)$$

To estimate how much variance of oxygenation the neural activity can predict, we first split the observed data into 2 segments with equal length. We then calculated the HRF using the first half of the observed data. We smoothed the HRF using a Savitzky–Golay filter (third order, 11-point frame length). Next, we convolved the HRF with the gamma-band LFP power from the second half and estimated the oxygenation predicted by neural activity using the following equation:

$$O_{2}=LFP⊗HRF+\varepsilon .$$

The efficacy of the prediction was quantified by calculating the correlation coefficient (R) between the prediction and actual oxygenation. The process was shown in **S4 Fig**.

#### Hemodynamic response function kernel fitting

To quantify the temporal features of HRF, the HRF for tissue oxygenation was fitted using 2 gamma-variate fitting processes [25,37,132–134] using 2 gamma-variate function kernels of the following form,

$$HRF(t,T_{i},W_{i},A_{i})=\sum_{i=1}^{2}A_{i}*{\left(\frac{t}{T_{i}}\right)}^{\alpha_{i}}*e^{\left(\frac{t-T_{i}}{-\beta_{i}}\right)},$$

where *α*_*i*_ = (*T*_*i*_/*W*_*i*_)^2^*8.0*log(2.0), $\beta_{i}={W_{i}}^{2}/\left(T_{i}*8.0*\log\left(2.0\right)\right)$. For modeling HRF using a gamma-variate function kernel, we used a downhill simplex algorithm minimizing the sum square difference between measured and predicted hemodynamics. The goodness of fit was quantified as $R^{2}=1-\frac{\sum {\left({HRF}_{actual}-{HRF}_{model}\right)}^{2}}{\sum {\left({HRF}_{actual}-\bar{HRF}\right)}^{2}}$, where $\bar{HRF}$ is the mean value of the actual HRF. The amplitude (A), time-to-peak (T), and full-width at half maximum (W) of the kernel were then calculated.

### Modeling RBC spacing effects on tissue oxygenation

We identified the location of each RBC using custom code written in MATLAB from line scan images using 2PLSM (**Fig 6A**). Data were first undergoing visual inspection of motion artifacts to determine if the quality was sufficient for reliable RBC detection. To calculate the power spectral density of RBCs train, we estimated using a function specifically for point processes (Chronux toolbox function: mtspectrumsegpb). To estimate the RBC interval distribution, we pooled all observed RBC intervals during rest from different animals (*n =* 9 mice) together to determine the probability density function (PDF). However, it has been reported that only a small number of segments (approximately 0.5%) experience a stall at any given instant in awake mice [102,144], which makes the observation of capillaries with a cessation of RBC flow challenging. It is also not practical to measure a large number of capillaries with a sufficiently long duration to characterize the temporal dynamics using 2PLSM. To avoid the bias in estimating the RBC spacing PDF due to the rare occurrence of “stall” events in our experiments, we also estimated the PDF of “stall” events using data from [144]. Combining these 2 PDFs, we estimated a new PDF of RBC intervals to generate a synthetic dataset (matlab function: normrnd). In awake mice, the capillary RBC velocity is between 0.3 and 1 mm/s [44,200,205,206]; to account in the RBC size (approximately 7 μm) [91] and to make the simple model more physiological relevant, we excluded RBC intervals smaller than 10 ms using a truncated normal distribution. As the observed consecutive RBC intervals are not totally random and have a power law exponent ranging from 0.6 to 1.4 (**Fig 6D**), we then introduced long-range autocorrelation using inverse Fourier transform [207].

Using the generated RBC train time series, we then simulated the oxygenation change inside the capillary and in the nearby brain tissue (**Fig 6E** and **6F**). In capillaries, RBCs travel in single file, separated by plasma gaps of variable lengths, so the capillary blood is not a temporally homogenous oxygen source to the surrounding tissue. We therefore assumed that (1) the tissue is primarily oxygenated by the nearest capillary; (2) the space between RBC and capillary wall is minimal and that the capillary wall does not hinder the transport of oxygen; therefore, the oxygen concentration profile is continuous between blood and tissue; (3) oxygen transport within the tissue is assumed to be solely by molecular diffusion and governed by Fick’s second law of diffusion; and (4) the rate of consumption of oxygen by the tissue surrounding the capillary is constant. Under these assumptions, over time, the level of oxygen within the tissue rises until the amount of oxygen lost by the passing cells converges to a quasi-steady level. At this quasi-steady state, the oxygen level in the proximity of the capillary fluctuates between a maximum reached just after the passage of a RBC and a minimum midway prior to the arrival of the next RBC (**Fig 6E** and **6F**). The amount of oxygen delivered by a RBC to the tissue slice is the summation of the oxygen mass gained and consumed within the tissue during its residence.

To keep the model tractable, the geometry of the erythrocytes was not considered (for a more detailed model, see [91]), and the erythrocyte was treated as a point-like oxygen source [78]. The oxygen tension for each RBC was set to be the same, and the diffusion of oxygen from RBC to plasma was simulated with an exponential decay kernel measured in previous experiments [93–96].

To model tissue oxygen responses, we simulated a vessel with 3 μm radius and a tissue cylinder of 20 μm radius using Krogh cylinder model.

$$PtO2=PwO2+\frac{CMRO2}{4\alpha_{t}D_{t}}(r^{2}-R^{2}-2{Rt}^{2}ln(\frac{r}{R}))$$

where, D_t_ = 2,800 μm^2^/s [208], α_t_ = 1.39 μM/mm Hg [209], CMRO2 = 3 μmole/cm^3^/min [210]. As the transit time of RBCs is much faster than the tissue response time, the observed oxygenation is further smoothed using the response time, which is given by $R^{2}ln(\frac{r}{R})/\left(2D_{t}\right)$ [83]. R is the outer radius, and r is the ratio of outer to inner radii. In this way, oxygen delivery from capillaries decays rapidly with distance. As the oxygen probe samples a small region around the tip, we averaged tissue oxygen data within 10 μm away from the location of the probe. Finally, to account for the response time of the polarographic oxygen electrodes [20], we smoothed the averaged oxygen trace with a low-pass filter.

### Power spectral density and power law exponent

In the present study, we used a widely used power spectrum analysis for 1/f-like dynamics estimation in both brain hemodynamics and electrophysiology [8,68,69,75,211,212]. The power spectrum density (PSD) was obtained using the multitaper technique [114]. We tried to fit the power spectrum of oxygen/electrophysiology signal with a power law distribution using ordinary least squares regression (without additional weighting in the fitting algorithm). However, when linearly spaced frequency bins are considered under a logarithmic scale, bins in higher frequencies become progressively denser and thus gain disproportionate weight with respect to lower-frequency bins in a subsequent linear regression. To avoid this potential bias, we upsampled the PSD curve with logarithmically spaced frequency bins, resulting in equally spaced frequency bins under logarithmic scale, required to properly estimate the spectral exponent. We then used the simple ordinary least squares regression to the resampled PSD in order to increase comparability to other studies [8,68,69,75,211].

### Detrended fluctuation analysis

Although a power law fit can provide relatively good fit to the brain oxygenation power spectrum, to rigorously test the hypothesis that brain hemodynamic signals are 1/f-like, we also used a time-domain method, DFA [115], which complements the above frequency-domain approach.

The DFA procedure measures the amount of fluctuation F(n) of detrended integrated signal at different length scales, thereby revealing the scaling properties of the signal. The method calculates the fluctuation amplitude, F(n), as a function of time scale n. Specifically, for a time series {*x*_*i*_, *i* = 1,2,…,*N*}, DFA performs the following processes: (1) we removed the global mean and integrating the time series by $X_{t}=\sum_{i=1}^{t}\left(x_{i}-\bar{x}\right)$, where $\bar{x}$ denotes the mean values of the time series *x*_*i*_; (2) we divided the integrated signal *X*_*t*_ into nonoverlapping windows of the same chosen size n; (3) we detrended the integrated signal *X*_*t*_ in each window using polynomial functions to obtain residuals by $\hat{X_{t}}=X_{t}-Y_{t}$, where *Y*_*t*_ denotes the trend obtained by polynomial fit and $\hat{X_{t}}$ denotes the integrated time series after detrending; and (4) we calculated the root mean square of residuals in all windows as detrended fluctuation amplitude using $F(n)=\sqrt{\frac{1}{N}\sum_{t=1}^{N}{\hat{X_{t}}}^{2}}$. The same steps above are repeated for different time scales n. The second order of polynomial function was used to detrend data in step 3 to eliminate the effect of possible linear trends in original data. A power law form of F(n), where F(n)∝*n*^*α*^, indicates a 1/f-like structure in the fluctuations. The parameter *α*, called the scaling exponent, quantifies the temporal correlation as follows [126,127]: if *α* = 0.5, there is no correlation in the fluctuations (“white noise”); if *α*>0.5, there are positive correlations, where large values are more likely to be followed by large values (and vice versa); if *α*<0.5, there are negative correlations, where large values are more likely to be followed by small values, and vice versa. Notably, the DFA method has the particular advantage of being applicable to both stationary and nonstationary data.

### Goodness of fit test

The goodness of fit was quantified with coefficients of determination (R^2^).

### Alternative model comparisons

Independent of whether power law model is a statistically good model, nevertheless, its non-power law alternatives may be a better model. To verify this, we compared the fit of alternative models, specifically, an exponential distribution and a log-normal distribution, to the power spectrum and DFA scaling results of different signals. We compared these models using Akaike information criterion (AIC), which is a common approach for selecting the best model among a set of fitted models. If all the models in the set assume normally distributed errors with a constant variance, then AIC can be easily computed from least squares regression statistics as

$$AIC=n\;\log\left({\hat{\sigma }}^{2}\right)+2K,$$

where ${\hat{\sigma }}^{2}=\frac{\sum {\hat{\epsilon }}_{i}^{2}}{n}$, and ${\hat{\epsilon }}_{i}^{2}$ are the estimated residuals for a particular candidate model. ${\hat{\sigma }}^{2}$ is the maximum likelihood estimation of the sum of squared residuals *σ*^2^, K is the total number of estimated regression parameters, including the intercept and *σ*^2^. As we have relatively small dataset, we used a second-order variant of AIC (AICc) to compare models: ${AIC}_{c}=AIC+\frac{2K(K+1)}{n-K-1}$. All alternative models used here have exactly 3 parameters, i.e., slope, intercept, and error term.

For each dataset, we compared the power law model’s AICc score with the AICc score of each alternative distribution, deriving ΔAIC_c_. Following standard practice, if ΔAIC_c_< 2, we conclude that there is little or no statistical evidence that the models fit the data differently. In this case, we say that the comparison is inconclusive and cannot distinguish between the 2 models. Otherwise, when ΔAIC_c_> = 2, we conclude that the model with the lower AICc value provides the better fit to the data.

### Evaluating analysis methods on synthetic data with ground truth

To demonstrate the suitability of power spectral analysis and DFA as a method to estimate power law scales of the same length as the signals used in our analysis, as well as the goodness of fit paradigm, we simulated time series with a stochastic Gaussian process of known long-range temporal dependence (fractional Gaussian noise). The power spectra of different types of signals are shown in **S1 Fig**. White noise (**S1 Fig**, panel A) has a flat power spectrum whose slope is near 0. Periodic noise (**S1 Fig**, panel B) shows a flat spectrum, with the exception of a large bump at 0.2 Hz, the center frequency of the large oscillations. In contrast, 1/f-like noise, generated using a circulant embedding method [213], shows power decreasing with frequency when the power spectrum is plotted on a log–log scale (**S1 Fig**, panel C). Summation of a periodic signal with a 1/f-like signal produces a hybrid spectrum (**S1 Fig**, panel D).

### Control recordings and analyses

Because fluctuations of resistivity in electronic conducting materials also exhibit 1/f noise [120,121], it is important to demonstrate that our data were not contributed by instrument noise. To address this, we measured PtO_2_ in one mouse postmortem using the same experimental setup. The power spectrum of these recordings had flat slope, characteristic of white noise (**Fig 1C**). This is further confirmed by DFA analysis (**Fig 1D**).

### Statistical analysis

Statistical analysis was performed using MATLAB. All summary data were reported as the mean ± SD unless stated otherwise. For visual representation of the data, we utilized box-and-whiskers plot (MATLAB function: boxplot) to illustrate the spread and differences of samples. The box shows the median ± interquartile range, and the whiskers show the data point that is no more than 1.5 times interquartile range (i.e., Tukey style). We also plotted sample mean to communicate more information about the dataset. Normality of the samples were tested before statistical testing using Anderson–Darling test (MATLAB function: adtest). For comparison of multiple populations, the assumption of equal variance for parametric statistical method was also tested (MATLAB function: vartest2 and vartestn). If criteria of normality and equal variance were not met, parametric tests (*t* test and one-way ANOVA) were replaced with a nonparametric method (Mann–Whitney *U* test, Wilcoxon signed-rank test, and Kruskal–Wallis ANOVA). All *p* values were Bonferroni corrected for multiple comparisons. Significance was accepted at *p* < 0.05.

## Supporting information

S1 Fig Illustration of white noise, periodic, and 1/f-like signal. (**A**) An example of white noise (left), its power spectrum (middle), and DFA results (right). The solid black line in the middle and right subplots denotes the ordinary least squares regression fit. (**B**) An example of a periodic signal with peak frequency centered at 0.2 Hz (left), its power spectrum (middle), and DFA results (right). The DFA scaling is clearly deviated from a linear fit. (**C**) An example of fractional Gaussian noise (i.e., 1/f-like) with Hurst exponent = 0.9 (left), its power spectrum (middle), and DFA results (right). (**D**) An example of additive signal combining fractional Gaussian noise and periodic signal (left), its power spectrum (middle), and DFA results (right). The data used to generate this figure are available at https://doi.org/10.5061/dryad.pg4f4qrmt. DFA, detrended fluctuation analysis.
(EPS)

S2 Fig 1/f-like power spectra of broadband LFPs and spike rate. (**A**) Experimental setup. Top, schematic showing all laminar electrophysiology measurement sites in FC (*n =* 4 sites) and FL/HL (*n* = 6 sites). Bottom, schematic showing the layout of the electrodes and measurement depth. (**B**) An example trace showing the broadband LFP at different cortical depths in the FL/HL. (**C**) An example trace showing the MUA at different cortical depths in the FL/HL in the same trial as (**B**). (**D**) Top, power spectrum of broadband (1–100 Hz) LFP across cortical depth during rest (left) and periods including rest and locomotion (right). Bottom, power law fit exponent. (**E**) As (**D**) but for spike rate. For better visualization and comparison between these signals, the power spectrum curves in (**D**) and (**E**) have been vertically shifted between different cortical depths. The shaded area is shown as mean ± SEM in (**D**, top) and (**E**, top). Data in (**D**, bottom) and (**E**, bottom) are shown as median ± interquartile range using boxplot, with the sample mean shown as dashed lines. The data used to generate this figure are available at https://doi.org/10.5061/dryad.pg4f4qrmt. FC, frontal cortex; FL/HL, forelimb/hindlimb; LFP, local field potential; MUA, multiunit activity.
(EPS)

S3 Fig BLP in sub-alpha and beta frequency bands are not 1/f-like. Related to **Fig 2.** (**A**) Experimental setup. (**B**) Normalized (by total power between 0.01–1 Hz) power spectrum of the beta-band power of LFP at different cortical depths. Dashed line denotes the linear regression fit of the power. (**C**) As (**B**) but for sub-alpha band power of LFP. Data are shown as mean ± SEM in (**B**) and (**C**). For better visualization and comparison between these signals, the power spectrum curves in (**B**) and (**C**) have been vertically shifted between different cortical depths. (**D**) Group average (*n =* 9 sites) of power law exponent for power spectrum of BLP in beta frequency band across different cortical layers during periods of rest (left) and periods including both rest and locomotion (right). (**E**) As (**D**) but for BLP in sub-alpha frequency band. (**F**) Group average (*n* = 9 sites) of DFA scaling exponent across different cortical layers during periods of rest (left) and periods including both rest and locomotion (right). (**G**) As (**F**) but for BLP in sub-alpha frequency band. In (**D**–**G**), gray circles denote the measurements in FL/HL (*n* = 6 sites), while the orange circles denote the measurements in FC (*n* = 3 sites). Data in (**D**–**G**) are shown as median ± interquartile range using boxplot, with the sample mean shown as dashed lines. The data used to generate this figure are available at https://doi.org/10.5061/dryad.pg4f4qrmt. BLP, band-limited power; DFA, detrended fluctuation analysis; FC, frontal cortex; FL/HL, forelimb/hindlimb; LFP, local field potential.
(EPS)

S4 Fig Illustration of the deconvolution and convolution process. Related to **Fig 4.** To estimate how much variance of oxygenation the neural activity can predict, we first split the observed data (approximately 40 minutes) into 2 segments of equal length. We then calculated the HRF using the first half of the observed data. To increase the signal-to-noise level of the HRF, we smoothed the HRF using a Savitzky–Golay filter (third order, 11-point frame length). Next, we convolved the HRF with the gamma-band LFP power from the second half and estimated the oxygenation predicted by neural activity (orange line). HRF, hemodynamic response function; LFP, local field potential.
(EPS)

S5 Fig Impact of pharmacological silencing on neural dynamics. Related to **Fig 5.** (**A**) Experimental setup. (**B**) Normalized (by total power between 1–100 Hz) power spectrum of broadband LFP signal before (black) and after (red) application of CNQX/AP5/muscimol using data during rest (left) and using data including rest and locomotion (right). (**C**) Group average (*n* = 9 mice) of fitted power law exponent of broadband LFP. (**D**) Normalized (by total power between 0.01–1 Hz) power spectrum of gamma-band power of LFP before (black) and after (red) application of CNQX/AP5/muscimol using data during rest (left) and using data including rest and locomotion (right). (**E**) Group average (*n* = 9 mice) of power law exponent of BLP for gamma-band LFP. (**F**) As (**E**) but for DFA scaling exponent. Data are shown as mean ± SEM in (**B**) and (**D**). In (**C**), (**E**), and (**F**), gray circles denote measurements in FL/HL (4 mice), and orange circles denote measurements in FC (5 mice). Data in (**C**), (**E**), and (**F**) are shown as median ± interquartile range using boxplot, with the sample mean shown as dashed lines. The data used to generate this figure are available at https://doi.org/10.5061/dryad.pg4f4qrmt. AP5, (2R)-amino-5-phosphonopentanoic acid; BLP, band-limited power; CNQX, 6-cyano-7-nitroquinoxaline-2,3-dione; DFA, detrended fluctuation analysis; FC, frontal cortex; FL/HL, forelimb/hindlimb; LFP, local field potential.
(EPS)

S6 Fig Power spectrum and DFA of respiratory rate fluctuations. (**A**) Power spectrum of respiratory rate fluctuations before (black) and after (red) application of CNQX/AP5/muscimol using resting data (left) and data including both rest and locomotion (right). Data are shown as mean ± SEM. (**B**) Power law exponent of respiratory rate fluctuations before (black) and after (red) application of CNQX/AP5/muscimol using resting data (left) and data including both rest and locomotion (right). (**C**) As (**B**) but for DFA scaling exponent. *paired *t* test, t(8) = 3.5835, *p* = 0.0072. In (**B**) and (**C**), gray circles denote measurements in FL/HL (4 mice), and orange circles denote measurements in FC (5 mice). Data in (**B**) and (**C**) are shown as median ± interquartile range using boxplot, with the sample mean shown as dashed lines. The data used to generate this figure are available at https://doi.org/10.5061/dryad.pg4f4qrmt. AP5, (2R)-amino-5-phosphonopentanoic acid; CNQX, 6-cyano-7-nitroquinoxaline-2,3-dione; DFA, detrended fluctuation analysis; FC, frontal cortex; FL/HL, forelimb/hindlimb.
(EPS)

S1 Table Comparison of power law and alternative distributions fit. (DOCX)

S2 Table Goodness of fit for each physiological time series. (DOCX)

S1 Text Supplementary results. (DOCX)

## Abbreviations

aCSF: artificial cerebrospinal fluid
AIC: Akaike information criterion
AP5: (2R)-amino-5-phosphonopentanoic acid
BLP: band-limited power
BOLD: blood oxygenation level-dependent
CMRO_2_: cerebral metabolic rate of oxygen
CNQX: 6-cyano-7-nitroquinoxaline-2,3-dione
DFA: detrended fluctuation analysis
EAT: erythrocyte-associated transient
FC: frontal cortex
FL/HL: forelimb/hindlimb
fMRI: functional magnetic resonance imaging
HRF: hemodynamic response function
LFP: local field potential
MUA: multiunit activity
PDF: probability density function
PoRTS: polished and reinforced thin-skull
PSD: power spectrum density
RBC: red blood cell
SD: standard deviation
2PLSM: two-photon laser scanning microscopy
