# Supplementary material for: Origins of 1/f-like tissue oxygenation fluctuations in the murine cortex
Source: PLoS Biol. 2021 Jul 15;19(7):e3001298. doi: 10.1371/journal.pbio.3001298 (PMC8282088; doi:10.1371/journal.pbio.3001298)
Supplement: S1 Table — (DOCX) [file pbio.3001298.s007.docx]

**S1 Table. Comparison of power-law and alternative distributions fit**

|  |  | **Alternative** | **P ∝ f** | **Data segments** | **Test outcome** | | |
| --- | --- | --- | --- | --- | --- | --- | --- |
|  |  |  |  |  | Favor Power law | Inconclusive | Favor alternative |
| Brain tissue oxygenation | PSD | Exponential | ${ae}^{-bx}$ | Rest | 29.2% | 0.7% | 70.1% |
|  |  |  |  | All data | 89.9% | 0% | 10.1% |
|  |  | Log-normal | $\frac{1}{x}e^{-\frac{{(logx-u)}^{2}}{2\sigma^{2}}}$ | Rest | 27.1% | 0.7% | 72.2% |
|  |  |  |  | All data | 89.2% | 0% | 10.8% |
|  | DFA | Exponential | ${ae}^{-bx}$ | Rest | 100% | 0% | 0% |
|  |  |  |  | All data | 100% | 0% | 0% |
|  |  | Log-normal | $\frac{1}{x}e^{-\frac{{(logx-u)}^{2}}{2\sigma^{2}}}$ | Rest | 68.1% | 0.7% | 31.3% |
|  |  |  |  | All data | 83.8% | 0% | 16.2% |
| BLP at gamma-band | PSD | Exponential | ${ae}^{-bx}$ | Rest | 44.4% | 11.1% | 44.4% |
|  |  |  |  | All data | 100% | 0% | 0% |
|  |  | Log-normal | $\frac{1}{x}e^{-\frac{{(logx-u)}^{2}}{2\sigma^{2}}}$ | Rest | 33.3% | 55.6% | 11.1% |
|  |  |  |  | All data | 100% | 0% | 0% |
|  | DFA | Exponential | ${ae}^{-bx}$ | Rest | 100% | 0% | 0% |
|  |  |  |  | All data | 100% | 0% | 0% |
|  |  | Log-normal | $\frac{1}{x}e^{-\frac{{(logx-u)}^{2}}{2\sigma^{2}}}$ | Rest | 66.7% | 0% | 33.3% |
|  |  |  |  | All data | 100% | 0% | 0% |
