# Supplementary material for: Origins of 1/f-like tissue oxygenation fluctuations in the murine cortex
Source: PLoS Biol. 2021 Jul 15;19(7):e3001298. doi: 10.1371/journal.pbio.3001298 (PMC8282088; doi:10.1371/journal.pbio.3001298)
Supplement: S2 Table — (DOCX) [file pbio.3001298.s008.docx]

**S2 Table. Goodness-of-fit for each physiological time series**

| Signal | Goodness-of-fit (R^2^) | | | | Related information |
| --- | --- | --- | --- | --- | --- |
|  | Power-law exponent | | DFA scaling exponent | |  |
|  | Rest | All data | Rest | All data |  |
| Brain oxygenation, laminar distribution | 0.62±0.22 | 0.96±0.06 | 0.96±0.03 | 0.98±0.02 | **Fig 1**E-**1**G |
| Gamma-band BLP, laminar electrodes | 0.21±0.15 | 0.78±0.10 | 0.97±0.03 | 0.98±0.01 | **Fig 2**D-**2**F |
| Brain oxygenation, actual | N/A | 0.94±0.08 | N/A | 0.98±0.01 | **Fig 4**C-**4**E |
| Brain oxygenation, prediction | N/A | 0.82±0.12 | N/A | 0.98±0.02 |  |
| Brain oxygenation, aCSF | 0.56±0.19 | 0.96±0.04 | 0.96±0.02 | 0.98±0.01 | **Fig 5**D-**5**F |
| Brain oxygenation, CNQX/AP5/muscimol | 0.41±0.15 | 0.88±0.10 | 0.94±0.03 | 0.95±0.02 |  |
| Broadband LFP, laminar electrode | 0.91±0.04 | 0.55±0.16 | N/A | N/A | **S2 Fig**, panel D |
| MUA, laminar electrode | 0.73±0.14 | 0.86±0.12 | N/A | N/A | **S2 Fig**, panel E |
| Beta-band LFP, laminar electrode | 0.32±0.23 | 0.72±0.25 | 0.96±0.03 | 0.98±0.02 | **S3 Fig**, panels B, D and F |
| Subalpha-band LFP, laminar electrode | 0.31±0.21 | 0.77±0.13 | 0.97±0.02 | 0.99±0 | **S3 Fig**, panels C, E and G |
| Broadband LFP, aCSF | 0.96±0.02 | 0.88±0.07 | N/A | N/A | **S5 Fig**, panels B and C |
| Broadband LFP, CNQX/AP5/muscimol | 0.97±0.01 | 0.94±0.03 | N/A | N/A |  |
| Gamma-band BLP, aCSF | 0.26±0.25 | 0.78±0.13 | 0.98±0.02 | 0.98±0.01 | **S5 Fig**, panels D-F |
| Gamma-band BLP, CNQX/AP5/muscimol | 0.41±0.19 | 0.51±0.36 | 0.98±0.01 | 0.89±0.29 |  |
| Respiratory rate, aCSF | 0.41±0.13 | 0.87±0.11 | 0.96±0.02 | 0.99±0.00 | **S6 Fig** |
| Respiratory rate, CNQX/AP5/muscimol | 0.45±0.11 | 0.76±0.19 | 0.97±0.01 | 0.99±0.00 |  |
