# Supplementary material for: Origins of 1/f-like tissue oxygenation fluctuations in the murine cortex
Source: PLoS Biol. 2021 Jul 15;19(7):e3001298. doi: 10.1371/journal.pbio.3001298 (PMC8282088; doi:10.1371/journal.pbio.3001298)
Supplement: S1 Text — (DOCX) [file pbio.3001298.s009.docx]

**S1 Text. Supplementary results**

**1/f-like power spectra of broadband LFPs and spike rate.** Related to **S2 Fig**. For all cortical layers, the power spectrum of broadband LFPs showed 1/f-like behavior above 5 Hz, so we fit the power spectrum between 5-100 Hz at each layer with a power-law distribution to avoid the “shoulder”. To compare with the oxygen measurements, we selected LFPs from recordings 100 µm, 300 µm, 500 µm and 800 µm below the cortical surface. We found the fitted power law exponent was not significantly different across cortical depths at rest (2.09 ± 0.46 at 100 µm, 1.99 ± 0.39 at 300 µm, 1.98 ± 0.41 at 500 µm, 2.11 ± 0.55 at 800 µm; n = 6 mice (9 sites), one-way ANOVA, F(3,35) = 0.1912, p = 0.9016, **S2 Fig**, panel D). Including locomotion period did not change the relationship across cortical depths (1.82 ± 0.37 at 100 µm, 1.81 ± 0.20 at 300 µm, 1.76 ± 0.31 at 500 µm, 1.81 ± 0.40 at 800 µm; n = 6 mice (9 sites), one-way ANOVA, F(3,35) = 0.0571, p = 0.9817, **S2 Fig**, panel D). Compared to the resting only data, including the locomotion periods slightly decreased the power law exponent (average across all cortical layers for each animal, n = 6 mice (9 sites), rest: 2.04 ± 0.42, rest and locomotion: 1.80 ± 0.29, paired t-test, t(8) = 2.3421, p = 0.0473).

In addition to the broad-band LFPs, the spiking rate of neurons is also correlated with vasodilation, increases in blood flow and blood oxygenation [25, 30, 32, 38, 214]. To address whether the spike rate activity also shows 1/f-like activity, we estimated power spectrum of multi-unit spike trains after minimally smoothing the spike trains with a Gaussian kernel with full-width at half maximum (FWHM) of 10 ms. Fitting spike train power with a power-law distribution revealed 1/f-like dynamics at rest (0.46 ± 0.25 at 100 µm, 0.30 ± 0.19 at 300 µm, 0.42 ± 0.16 at 500 µm, 0.51 ± 0.31 at 800 µm; n = 6 mice (9 sites), one-way ANOVA, F(3,35) = 1.3417, p = 0.2782, **S2 Fig**, panel E), and using data including both rest and locomotion (0.41 ± 0.16 at 100 µm, 0.51 ± 0.28 at 300 µm, 0.36 ± 0.17 at 500 µm, 0.61 ± 0.32 at 800 µm; n = 6 mice (9 sites), Kruskal-Wallis test, $\chi^{2}$(3, 35) = 3.1842, p = 0.3641, **S2 Fig**, panel E). Compared to the resting only data, including the locomotion periods does not change the power law exponent (average across all cortical layers for each animal, n = 6 mice (9 sites), rest: 0.43 ± 0.09, rest and locomotion: 0.47 ± 0.16, paired t-test, t(8) = 1.0898, p = 0.3075). The fitted exponent of the resting power spectrum of spiking activity is much smaller compared to the broad-band LFPs (LFPs: 2.04 ± 0.42, MUA: 0.43 ± 0.09, Wilcoxon rank sum test, p < 0.0001), which is consistent with study [215] showing that 1/f scaling in the LFP power indicates the presence of step like transitions in the LFP trace and says little about properties of the associated neuronal firing.

**Power-law exponent and DFA scaling exponent of BLP of local field potential**. Related to **Fig 2** and **S3 Fig**. We calculated the power of beta-band (10-30 Hz) LFP oscillations and estimated the power law fitting exponent. In contrast to the broad-band LFPs (**S2 Fig**), a flat power spectrum was observed in the beta-band BLP in the frequency range below 1 Hz at rest (-0.03 ± 0.16 at 100 µm, 0.06 ± 0.16 at 300 µm, 0.09 ± 0.17 at 500 µm, 0.02 ± 0.16 at 800 µm, one-way ANOVA, F(3,34) = 0.9230, p = 0.4412, **S3 Fig**, panels B and D), which is characteristic of white noise (**S1 Fig**, panel A). Fitting all the data, including both rest and locomotion periods, significantly increased the power law exponent (rest: 0.03 ± 0.11, all data: 0.38 ± 0.16, paired t-test, t(8) = 5.7944, p < 0.0001), and showed no laminar difference (0.43 ± 0.17 at 100 µm, 0.39 ± 0.18 at 300 µm, 0.35 ± 0.21 at 500 µm, 0.37 ± 0.22 at 800 µm, one-way ANOVA, F(3,34) = 0.2424, p = 0.8661, **S3 Fig**, panels B and D).

We calculated the power of sub-alpha band (1-8 Hz) LFP oscillations and estimated the power law fitting exponent. In contrast to the broad-band LFPs, a flat power spectrum was observed in the sub-alpha band BLP in the frequency range below 1 Hz at rest (0.03 ± 0.16 at 100 µm, 0.03 ± 0.19 at 300 µm, 0.06 ± 0.13 at 500 µm, 0.06 ± 0.16 at 800 µm, one-way ANOVA, F(3,34) = 0.1171, p = 0.9494, **S3 Fig**, panels C and E), which shows a characteristic of white noise (**S1 Fig**, panel A). Fitting all the data, including both rest and locomotion periods, significantly increased the power law exponent (rest: 0.05 ± 0.12, all data: 0.48 ± 0.17, paired t-test, t(8) = 7.4288, p < 0.0001), and showed no laminar difference (0.55 ± 0.18 at 100 µm, 0.45 ± 0.20 at 300 µm, 0.51 ± 0.19 at 500 µm, 0.45 ± 0.21 at 800 µm, one-way ANOVA, F(3,34) = 0.5340, p = 0.6624, **S3 Fig**, panels C and E).

We further calculated the scaling exponent of band-limited power of LFP oscillations using DFA. For beta-band LFP oscillations, no significant differences were observed among different cortical depths using resting data (0.58 ± 0.07 at 100 µm, 0.61 ± 0.08 at 300 µm, 0.64 ± 0.08 at 500 µm, 0.63 ± 0.09 at 800 µm, one-way ANOVA, F(3,35) = 0.8882, p = 0.4577, **S3 Fig**, panel F), as well as using both rest and locomotion data (0.69 ± 0.08 at 100 µm, 0.69 ± 0.08 at 300 µm, 0.70 ± 0.09 at 500 µm, 0.68 ± 0.09 at 800 µm, one-way ANOVA, F(3,35) = 0.0678, p = 0.9766, **S3 Fig**, panel F). For sub-alpha band LFP oscillations, no significant differences were observed among different cortical depths using resting data (0.63 ± 0.09 at 100 µm, 0.68 ± 0.08 at 300 µm, 0.66 ± 0.09 at 500 µm, 0.67 ± 0.11 at 800 µm, one-way ANOVA, F(3,35) = 0.4192, p = 0.7404, **S3 Fig**, panel G), as well as using both rest and locomotion data (0.80 ± 0.12 at 100 µm, 0.75 ± 0.10 at 300 µm, 0.76 ± 0.14 at 500 µm, 0.75 ± 0.11 at 800 µm, Kruskal-Wallis test, $\chi^{2}$(3, 35) = 0.8138, p = 0.8462, **S3 Fig**, panel G). Fitting all the data, including both rest and locomotion periods, significantly increased the DFA scaling exponent for beta-band LFP oscillations (rest: 0.62 ± 0.07, all data: 0.69 ± 0.08, paired t-test, t(8) = 2.3356, p = 0.0477) and slightly increased the DFA scaling exponent for sub-alpha band LFP oscillations (rest: 0.66 ± 0.08, all data: 0.77 ± 0.11, paired t-test, t(8) = 2.1978, p = 0.0592).

**Suppressing neural activity affects power law exponent of broad band LFP.** Related to **S5 Fig**. We asked if this suppression of spiking and LFP oscillations changes the 1/f-like dynamics of neural activity. When the craniotomy was superfused with aCSF, the power spectrum of raw LFP was relatively flat in the lower frequency range (below a “knee” at 5 Hz), and the rest followed a form close to $P \propto1/{f^{2}}$. Application of CNQX/AP5/muscimol suppressed power at higher frequencies (5-100 Hz), but did not change the power law scaling at rest (aCSF, 2.18 ± 0.18; CNQX/AP5/muscimol, 2.12 ± 0.19; paired t-test, t(8) = 0.5458, p = 0.6001, **S5 Fig**, panels B and C). Including locomotion period did not change the relationship (aCSF, 1.92 ± 0.20; CNQX/AP5/muscimol, 2.08 ± 0.19; paired t-test, t(8) = 1.9318, p = 0.0895, **S5 Fig**, panels B and C). For lower frequency band (1-5 Hz), CNQX/AP5/muscimol application increased the power law scaling at rest (aCSF, -0.21 ± 0.30; CNQX/AP5/muscimol, 1.36 ± 0.73; Wilcoxon rank sum test, p < 0.0001). Including running period does not change the relationship (aCSF, -0.15 ± 0.28; CNQX/AP5/muscimol, 1.34 ± 0.69; Wilcoxon rank sum test, p < 0.0001).

We also calculated the frequency dependence of the band limited power fluctuations in the gamma-band of LFP (**S5 Fig**, panels D and E) during resting periods (aCSF: 0.13 ± 0.20; CNQX/AP5/muscimol: -0.12 ± 0.29, Wilcoxon rank sum test, p = 0.1135) and during resting and running period (aCSF: 0.54 ± 0.14; CNQX/AP5/muscimol: 0.29 ± 0.35, Wilcoxon rank sum test, p = 0.1903), and did not observe any change in terms of power-law scaling before and after drug application. DFA scaling exponents reproduce the results using power spectrum analysis. Suppression of neural activity did not change the DFA scaling exponent of band-limited power of Gamma-band LFP (**S5 Fig,** panel F) using rest data (aCSF: 0.57 ± 0.07; CNQX/AP5/muscimol: 0.51 ± 0.09; n = 9 mice, paired t-test, t(8) = 1.5092, p = 0.1697) and entire dataset (aCSF: 0.68 ± 0.06; CNQX/AP5/muscimol: 0.56 ± 0.17; n = 9 mice, Wilcoxon rank sum test, p = 0.1359).

**Comparison of alternative models**. Related to **S1 Table**. We compared the fit of alternative models, specifically, an exponential distribution and a log-normal distribution, to the power law fit of the power spectrum and DFA. For resting data, AIC tests find only modest support for the power-law distribution over two alternatives. The exponential distribution is favored over the power law (70.1%) more often than vice-versa (29.2%) for resting power spectrum. Similarly, the log-normal is a broad distribution that can exhibit heavy tails, but which is nevertheless not power-law. The log-normal is favored (72.2%) over the power law (27.1%) for resting power spectrum. However, analysis using entire dataset strongly favor (~90% of cases) the power-law model fit over alternative models. For DFA results, a power-law model fit was also strongly favored.

**Comparison of goodness-of-fit**. Related to **S2 Table**. As both methods, either power spectrum analysis or detrended fluctuation analysis, depend on line fitting, our results may be affected by the fitting quality. To make sure that our data is sufficient linear across a wide range of frequency or time scales, we calculated the goodness-of-fit using coefficient of determination (R^2^). For brain tissue oxygenation (**Fig 1**), the power-law fit is a good fit for power spectrum using only resting data (R^2^ = 0.62 ± 0.22), and a superior fit for power spectrum using both rest and locomotion data (R^2^ = 0.96 ± 0.06). For DFA analysis, a power-law fit provides almost perfect fit to the data. In addition, the observed increases in the power-law exponent and DFA scaling exponent of brain tissue oxygenation after suppressing neural activity (**Fig 5**) were not due to a change in fitting quality.

**Supplementary references**

214. Ma Y, Shaik MA, Kozberg MG, Kim SH, Portes JP, Timerman D, et al. Resting-state hemodynamics are spatiotemporally coupled to synchronized and symmetric neural activity in excitatory neurons. Proc Natl Acad Sci U S A. 2016;113(52):E8463-E71. pmid: 27974609.

215. Baranauskas G, Maggiolini E, Vato A, Angotzi G, Bonfanti A, Zambra G, et al. Origins of 1/f2 scaling in the power spectrum of intracortical local field potential. J Neurophysiol. 2012;107(3):984-94. pmid: 22090461.
